# Screening and characterization of novel specific peptides targeting MDA-MB-231 claudin-low breast carcinoma by computer-aided phage display methodologies

**DOI:** 10.1186/s12885-016-2937-2

**Published:** 2016-11-14

**Authors:** Franklin L. Nobrega, Débora Ferreira, Ivone M. Martins, Maria Suarez-Diez, Joana Azeredo, Leon D. Kluskens, Lígia R. Rodrigues

**Affiliations:** 1 Centre of Biological Engineering (CEB), University of Minho, Campus de Gualtar, 4710-057 Braga, Portugal; 2 Laboratory of Systems and Synthetic Biology, Wageningen University and Research Centre, Stippeneng 4, 6708WE Wageningen, The Netherlands

**Keywords:** Claudin-low breast cancer, Phage display, MDA-MB-231, PRWAVSP, DTFNSFGRVRIE

## Abstract

**Background:**

Claudin-low breast carcinoma represents 19% of all breast cancer cases and is characterized by an aggressive progression with metastatic nature and high rates of relapse. Due to a lack of known specific molecular biomarkers for this breast cancer subtype, there are no targeted therapies available, which results in the worst prognosis of all breast cancer subtypes. Hence, the identification of novel biomarkers for this type of breast cancer is highly relevant for an early diagnosis. Additionally, claudin-low breast carcinoma peptide ligands can be used to design powerful drug delivery systems that specifically target this type of breast cancer.

**Methods:**

In this work, we propose the identification of peptides for the specific recognition of MDA-MB-231, a cell line representative of claudin-low breast cancers, using phage display (both conventional panning and BRASIL). Binding assays, such as phage forming units and ELISA, were performed to select the most interesting peptides (i.e., specific to the target cells) and bioinformatics approaches were applied to putatively identify the biomarkers to which these peptides bind.

**Results:**

Two peptides were selected using this methodology specifically targeting MDA-MB-231 cells, as demonstrated by a 4 to 9 log higher affinity as compared to control cells. The use of bioinformatics approaches provided relevant insights into possible cell surface targets for each peptide identified.

**Conclusions:**

The peptides herein identified may contribute to an earlier detection of claudin-low breast carcinomas and possibly to develop more individualized therapies.

**Electronic supplementary material:**

The online version of this article (doi:10.1186/s12885-016-2937-2) contains supplementary material, which is available to authorized users.

## Background

Breast cancer is the most frequent cancer among women, representing 25% of all cancer cases, and the most frequent cause of cancer death in less developed countries and the second in developed regions [1].

Breast cancer has long been recognized as a heterogeneous disease [2], challenging an effective detection, diagnosis and treatment. Initially based on morphological observations, this heterogeneity has been confirmed by high-throughput methods such as molecular profiling with microarrays. These have allowed the identification of specific biomarkers whose presence or absence enable distinguishing breast cancers into different subtypes. The currently accepted biomarkers include the estrogen (ER), progesterone (PR) and human epidermal growth factor 2 (HER2) receptors [3], diving breast cancer into the following subtypes: luminal A (ER^+^, PR^+/−^, HER2^−^), luminal B (ER^+^, PR^+/−^, HER2^+^), HER2 (ER^−^, PR^−^, HER2^+^), basal-like (ER^−^, PR^−^, HER2^−^) and claudin-low (ER^−^, PR^−^, HER2^−^) [4, 5]. The claudin-low subtype was initially clustered together with the basal-like but the presence of unique features (e.g., downregulation of claudin-3 and claudinin-4 and the low expression of proliferation marker Ki67) led to its own subtype [6, 7].

Each cancer subtype has a different prognosis and treatment response [4]. Luminal A and luminal B subtypes, characterized by the presence of ER, are commonly treated with hormone therapy with a good overall outcome; HER2 subtype, with the presence of HER2 can be treated with anti-HER2 monoclonal antibody therapy; but the basal-like and claudin-low subtypes, due to the absence of expression of a recognizable therapeutic target, lack targeted therapeutic options [8, 9]. Unfortunately, these represent about 19% of all breast cancer cases and include those with worst prognosis due to its aggressive and metastatic nature and high rates of relapse [10]. The identification of specific molecular biomarkers for these subtypes would be a valuable contribution to a more precise diagnosis and to the development of individualized therapies to different molecular subgroups.

However, the quest for molecular biomarkers specific for cancer cells remains a challenge due to the lack of affinity reagents that can specifically bind to unique molecular targets on the surface of the these cells. The isolation and identification of such reagents is vital for clinical applications in cancer diagnosis and therapy [11]. Evolutionary screening techniques, such as phage display [12], have demonstrated incredible capacity to identify affinity reagents for a wide variety of targets (proteins, nucleic acids, inorganic materials, cells, among others) [13, 14]. In fact, phage display has already been used to generate recombinant antibody fragments that specifically recognize breast cancer subpopulations [15], as well as cell-targeting peptides for SK-BR-3 breast cancer cells [16]. In addition, phage display does not require prior knowledge of the cell surface, has low costs, and the cell-specific peptides identified typically present low immunogenicity [17, 18].

In this work, we used phage display to identify peptides specifically recognizing the claudin-low breast cancer cell line MDA-MD-231. The identification of such peptides could open new perspectives for the development of targeted therapies against this specific breast cancer subtype. Binding assays were performed to select the most specific peptides and a bioinformatics analysis was implemented to evaluate their potential targets on the cell surface.

## Methods

### Library diversity and preparation

The M13KE phage and its host, *Escherichia coli* ER2387, were obtained from New England Biolabs (NEB). Two different libraries of M13KE were used, namely a home-made 7-mer library and a commercial 12-mer library from NEB (E8110S). The construction of the 7-mer library was performed as described in [19], using primers 5′–CATGCCCGGGTACCTTTCTATTCTC–3′ and 5′– (NNN)_7_AGAGTGAGAATAGAAAGGTACCCGGG–3′ and digested as in the protocol for M13KE DNA insertion (7.2 kb).

### Cell line and culture

The human cancer cell lines MDA-MB-231 (claudin-low subtype), SK-BR-3 (HER2 subtype), Hs 578 T (basal-like subtype) and MDA-MB-435 (melanoma [20]) were kindly provided by the Institute of Molecular Pathology and Immunology at the University of Porto (IPATIMUP). The human mammalian cell line MCF-10-2A (ATCC CRL-10781) is non-tumorigenic and was used as a control. MDA-MB-231, SK-BR-3, Hs 578 T, and MDA-MB-435 cells were routinely cultured in Dulbecco’s Modified Eagle Medium (DMEM, Biochrom) supplemented with 10% (v/v) fetal bovine serum (FBS, Biochrom) and 1% (v/v) penicillin-streptomycin (Biochrom). MCF-10-2A cells were grown in a 1:1 solution of DMEM and HAM’s F-12 medium supplemented with 5% horse serum (Merck Millipore), 20 ng.mL^−1^ epidermal growth factor (Merck Millipore), 100 ng.mL^−1^ cholera toxin (Sigma-Aldrich), 0.01 mg.mL^−1^ insulin (Sigma-Aldrich), 500 ng.mL^−1^ hydrocortisone, 95% (Sigma-Aldrich) and 1% penicillin-streptomycin. All cell lines were cultured at 37 °C and 5% CO_2_. Subculturing was performed at 80% confluence, by washing the monolayer with sterile phosphate buffered-saline (PBS), pH 7.4, without Ca^2+^ and Mg^2+^, and detaching the cells with Trypsin/EDTA solution 0.05%/0.2% (w/v) (Biochrom). The cell suspension was centrifuged at 250 × *g* for 7–10 min and the cell pellet was resuspended on fresh growth medium, counted and split according to the experimental needs.

### Panning experiments – conventional selection versus BRASIL

Both conventional phage display and BRASIL [21] methods were used to compare their performance in the selection of a peptide specific to the MDA-MD-231 cells. The BRASIL method is in principle faster than the conventional panning and by using counter-selection it reduces the number of false positives. However, this methodology uses cells in suspension, which may hide surface receptors that are only available in the adherent state. The panning experiments with both methodologies were performed equally for the 7-mer and the 12-mer libraries. The experimental setting can be seen in Additional file 1: Table S1.

#### Conventional selection (surface panning procedure – direct target coating)

One mL of MDA-MB-231 cell suspension at a concentration of 10^6^ cells.mL^−1^ was added to a 6-well microtiter plate and incubated overnight at 37 °C in a 5% CO_2_ humidified incubator. The medium was then removed and the wells completely filled with blocking buffer (0.1 M NaHCO_3_ (pH 8.6, Sigma), 5 mg.ml^−1^ Bovine Serum Albumin (BSA) (Sigma) solution IgG-free, low endotoxin suitable for cell culture (Sigma). After an incubation of 1 h at 4 °C, the blocking solution was discarded and the wells washed 6 times with Tris Buffered Saline with Tween-20 (TBST, TBS with 0.1% (v/v) Tween-20) (Sigma-Aldrich). One mL of a 100-fold dilution in TBST of the library (7-mer or 12-mer) (1x10^11^ for a library with 2x10^9^ clones) was added to the coated wells and rocked gently for 60 min at 4 °C (to limit phage internalization). The non-binding phage was discarded and the wells were washed 10 times with TBST. The bound phage was then eluted with 750 μL of PBS 1x (137 mM NaCl, 2.7 mM KCl, 10 mM Na_2_HPO_4_ and 1.8 mM KH_2_PO_4_), and rocked gently for 60 min at 4 °C. The eluate was transferred to a microcentrifuge tube and the titer was determined using the double layer agar technique [22] in LB plates containing 100 μM IPTG and 20 μg.mL^−1^ X-gal, counting the blue colonies. The remaining eluate was amplified by adding the eluate to 20 mL early-log ER2738 culture and incubating with vigorous shaking for 4.5 h at 37 °C. The culture was spun at 12,000 × *g* for 10 min at 4 °C, and the supernatant was transferred to a fresh tube and re-spun. The upper 80% of the supernatant was transferred to a new tube and the phage was precipitated with 1/6 volume of 20% polyethylene glycol (PEG) 8000/2.5 M NaCl for at least 2 h at 4 °C. This solution was centrifuged at 12,000 × *g* for 15 min at 4 °C, the supernatant was discarded and the phage pellet was suspended in 1 mL TBS. PEG/NaCl precipitation was repeated and the final pellet suspended in 200 μL TBS. The titer was determined as previously described. The whole process was repeated for a total of 8 rounds of panning.

A control panning experiment was carried out using streptavidin as the target, including 0.1 μg.mL^−1^ streptavidin in the blocking solution. The bound phage was eluted with 0.1 mM biotin in TBS for at least 30 min. After 3 rounds of enrichment/amplification, the consensus sequence for streptavidin-binding peptides was assessed to confirm the inclusion of the motif His-Pro-Gln.

#### BRASIL

A biopanning protocol was used as described in [21]. Briefly, MDA-MB-231 cells.mL^−1^ were collected, centrifuged (250 × *g*, 10 min) and the pellet suspended in 1 mL of complete DMEM medium, containing 1% (w/v) BSA. The solution was centrifuged and this step repeated 3 times; the cells were re-suspended in complete growth medium containing 3% (w/v) BSA solution and kept on ice. Ten μL of the phage library (7-mer or 12-mer) were added to the previous cell suspension and incubated on ice for 4 h. A bubble of 300 μL PBS was formed on a non-miscible organic phase (cyclohexane:dibutyl phthalate (1:9, v/v, Sigma)), and 200 μL of the cell suspension incubated with the phage library were gently inserted into the bubble. After centrifuging at 10,000 × *g* for 10 min, the pellet was recovered and washed with 50 μL Tris–HCl (10 mM, pH 9.5). Eluted phages were amplified between rounds using *E. coli* ER2738, purified and concentrated with 20% PEG 8000/2.5 M NaCl. Phage titer was determined as described above. The amplified phages were used for additional rounds of biopanning in a total of eight. A final round of counter-selection with MCF-10-2A cells (non-tumorigenic) was performed, differing from the previous rounds in the fraction collected, which in this case was the aqueous phase containing the phages that did not bind to the control cells.

### Preliminary analysis of the specificity and selectivity of a phage pool

#### Flow cytometry analysis

To characterize pool specificity and selectivity, the last round of the 12-mer phage pool from conventional panning was conjugated with Alexa 488 and analyzed using flow cytometry to evaluate the binding to MCF-10-2A (control, non-tumorigenic cells), MDA-MB-231, MDA-MB-435, SK-BR-3 and Hs 578 T cell lines. Briefly, 1×10^5^ cells were harvested, washed in PBS and blocked using PBS with 3% BSA at 4 °C for 1 h. Subsequently, the cells were washed with PBST 1× (PBS with 0.1% (v/v) Tween-20) and were incubated with 100 μL of fluorescent phage particles. The cells were rinsed again with PBST 1x and finally resuspended in 200 μL of PBS for flow cytometry analysis using a EC800™ flow cytometer analyzer (Sony Biotechnology Inc.) counting 20,000 events.

#### Tissue section analysis

For immunohistochemical analysis, serial sections of paraffin-embedded 231 mammary cancer tissue sections, kindly provided by Dr. João Nuno Moreira (CNC, Coimbra, Portugal), were treated as described in [23]. To maximize antibody binding, antigen retrieval was performed by heating the slides in 10 mM sodium citrate buffer (pH 6.0) at 95 °C for 20 min and the slow cooling at room temperature in the same buffer for about 20 min. Tissues were maintained humid at all time. Tissue sections were blocked using a 5% BSA solution and were incubated at room temperature for 30 min. Immunostaining was performed by adding 100 μL of the last round of the 12-mer phage pool (10^9^ PFUs.mL^−1^) to the tissue overnight at 4 °C [24, 25]. Sections were washed 4 times in TBST 1x for 5 min and 100 μL of the primary antibody rabbit anti-fd bacteriophage (working dilution of 1:5000 in BSA 1%), was added and incubated at 4 °C overnight. Sections were washed several times with TBST 1x and were challenged with the fluorescein isothiocyanate (FITC)-labelled goat anti-rabbit IgG secondary antibody (working dilution of 1:40 in 1% BSA) for 2 h at room temperature. After additional washing of the sections with TBST buffer, sections were counterstained with 4′, 6 - diamidino-2-phenylindole (DAPI, Vector Laboratories) for nuclear labelling and were mounted with Vectashield® mounting medium (Vector Laboratories). The tissue sections were allowed to dry for 1 h at room temperature in the dark and were sealed with nail polish. Images of the slides were captured using an Olympus BX51 microscope incorporated with a high-sensitivity camera Olympus DP71 with 60× magnification.

### Selection and screening of cell-specific peptides

#### Preparation of individual clones for peptide analysis

Single-stranded DNA (ssDNA) was prepared according to the standard protocol described in [19], using iodide buffer (10 mM Tris–HCl, 1 mM EDTA and 4 M NaI (Sigma-Aldrich), pH 8.0) and ethanol precipitation. The DNA pellet was suspended in 30 μL TE buffer (10 mM Tris–HCl, 1 mM EDTA, pH 8.0), quantified using Nanodrop 1000 and confirmed by 2% gel electrophoresis in SGTB (GRISP) buffer 1× at 200 V for 30 min.

#### PCR and confirmation electrophoresis

The insert sizes of the individual clones, as well as of the complete library were assessed by PCR using the forward primer 5′-TTAACTCCCTGCAAGCCTCA-3′ and the reverse primer 5′-CCCTCATAGTTAGCGTAACG -3′. PCR reactions were carried out using KAPA *Taq* polymerase in 20 μl reaction volume, containing 2 μL of phage DNA. The PCR conditions were the following: 25 cycles of denaturation at 95 °C for 30 s; annealing in the temperatures range from 45 to 70 °C, for 30 s; and extension at 72 °C for 30 s. Amplification was confirmed by 2% gel electrophoresis in SGTB buffer 1× at 200 V for 30 min.

#### DNA sequencing and insert analysis

The DNA products obtained were prepared for sequencing using Illustra ExoProStar 1-Step (GE Healthcare) and sent to Macrogen Inc. service using the M13-PIII sequencing primer 5′- TTAACTCCCTGCAAGCCTCA-3′, provided with the Ph.D.12-mer library kit for forward reading and the primer 5′ -CCCTCATAGTTAGCGTAACG-3′ for reverse reading. The Vector NTI Advance 11.5.0 software (Invitrogen – Life Technologies) was used for the analysis of correct insertion of the peptides taking into account that the displayed peptides are expressed at the N-terminus of pIII, followed by a short spacer (Gly-Gly-Gly-Ser) and then the wild-type pIII sequence.

### Binding assays

#### Binding assay with counting of blue colony forming units (pfu)

The binding of the peptides displayed on M13KE phage was evaluated following a procedure similar to the conventional panning. First, the individual clones were amplified, centrifuged at 12,000 × *g* for 10 min at 4 °C, and the supernatant used for phage concentration with 20% PEG 8000/2.5 M NaCl. Phages were suspended in 50 μL TBS and the titer was determined using the double layer agar technique. Then, 1 mL of MDA-MB-231 cells at a concentration of 10^6^ cells.mL^−1^ was added to a 6-well microtiter plate and incubated overnight at 37 °C and 5% CO_2_. MDA-MB-435 cells were used as a negative control in the same conditions. The cell medium was removed and the wells were washed 6 times with TBST. Then, 1 mL of each M13KE-peptide suspension, at a concentration of 1×10^11^ PFU.ml^−1^ was added to the wells and incubated for 60 min at 4 °C. The non-binding phage was discarded and the wells were washed 10 times with TBST. The bound phages were then eluted with 750 μL of PBS 1x and rocked gently for 60 min at 4 °C. The eluate was collected and the titer was determined using the double layer agar technique in IPTG/X-gal plates.

#### ELISA with direct target coating

ELISA was performed to rapidly determine whether a selected phage clone binds the target, using the protocol described in the NEB Phage Display manual [19]. For each clone to be characterized, one row of coated (with target cells) and uncoated wells were used. Plates were read at 405 to 415 nm (Promega Glomax 20/20 luminometer) and the signals (RLUs) obtained with and without target protein (cells) were compared.

### Bioinformatics analysis

#### Library analysis

Sequence similarities between the peptides obtained in this work and peptides reported in the literature targeting cancer cells (see Additional file 2: Table S3) were scored using Blosum45 matrices and the Needleman-Wunsch algorithm as implemented by the pairwise alignment function from the R *Biostrings* package version 2.38.2 [26]. The symmetric matrix containing the scores for the pairwise sequence alignments, *SC(i,j),* was converted into a similarity matrix taking into account the background values for each sequence following a procedure similar to the Context Likelihood of Relatedness (CLR) algorithm used to detect spurious association in transcriptional or metabolite association networks [27, 28]. Briefly, the likelihood of *SC(i,j)* is estimated using a null model given by considering all the alignment scores involving independently sequences *i* and *j*, *SC*_*i*_ and *SC*_*j*_, respectively. The background score is approximated as a joint normal distribution with *SC*_*i*_ and *SC*_*j*_ treated as independent variables. The final form of the likelihood estimate is:

1 $$ f\left({z}_i,{z}_j\right)=\sqrt{z_i^2}+{z}_j^2 $$

where

2 $$ {z}_i= max\left(0,\ \frac{SC\left(i,k\right) - {\mu}_i}{\sigma_i}\ \right) $$

and *μ*_*i*_ and *σ*_*i*_ are, respectively, the mean and the standard deviation of the empirical distribution of *SC(i, k)* with *k = 1,…,n*, and *n* the total number of considered sequences. The similarity estimate is then a matrix with entries *f(z*_*i*_ *, z*_*j*_ *)*. The similarity estimate was normalized, through dividing by its highest values, to use in Multidimensional scaling (MDS) plots, clustering and heatmap reconstruction using the R *gplots* library [29].

#### Docking studies

Known biomarkers of breast cancer were selected from a literature and databases search (see Additional file 3: Table S4). The biomarkers found were retrieved through the Kyoto Encyclopedia of Genes and Genomes (KEGG) for pathways and function analysis of biomarkers, Uniprot for protein characterization and amino acid sequences, GenBank for gene sequences, and Protein Data Bank (PDB) for tri-dimensional protein structures [30]. When protein structures were not available, they were predicted using the PHYRE2 software [31] and the peptide structures were predicted using PEPstrMOD [32, 33]. The resulting pdb files were used in a protein-peptide analysis performed using ClusPro 2.0 [34, 35] in all available models, by the peptide sequences identified by phage display against the tri-dimensional structures of the breast cancer biomarkers. Weighted score (*E*) was obtained by:

3 $$ E=0.40{E}_{\mathrm{rep}}+-0.40{E}_{\mathrm{att}}+600{E}_{\mathrm{elec}}+1.00{E}_{\mathrm{DARS}}\left(\mathrm{Balanced}\kern0.5em \mathrm{coefficients}\right) $$

4 $$ E=0.40{E}_{\mathrm{rep}}+-0.40{E}_{\mathrm{att}}+1200{E}_{\mathrm{elec}}+1.00{E}_{\mathrm{DARS}}\left(\mathrm{Electrostatic}\hbox{-} \mathrm{favored}\kern0.5em \mathrm{coefficients}\right) $$

5 $$ E=0.40{E}_{\mathrm{rep}}+-0.40{E}_{\mathrm{att}}+600{E}_{\mathrm{elec}}+2.00{E}_{\mathrm{DARS}}\left(\mathrm{Hydrophobic}\hbox{-} \mathrm{favored}\kern0.5em \mathrm{coefficients}\right) $$

6 $$ E=0.40{E}_{\mathrm{rep}}+-0.10{E}_{\mathrm{att}}+600{E}_{\mathrm{elec}}+0.00{E}_{\mathrm{DARS}}\left(\mathrm{Vand}\kern0.5em \mathrm{d}\mathrm{e}\mathrm{r}\kern0.5em \mathrm{Waals}\kern0.5em \mathrm{and}\kern0.5em \mathrm{Electrostatic}\kern0.5em \mathrm{coefficients}\right) $$

where the lowest energy state represents the highest binding. The tri-dimensional model structures obtained were visualized using UCSF Chimera version 1.10.2 [36]. Alignments were scored using Blosum45, 50 and 62 matrices.

### Statistical analysis

GraphPad Prism 5.03 (GraphPad Software, Inc.) was used for statistical analysis of the data. The significance of differences was evaluated using the One-way ANOVA with Tukey’s Multiple Comparison Test, considering a significance level of 95%.

## Results

### Identification by phage display of a peptide that recognizes the breast cancer cell line MDA-MB-231

Phage display search of ligands specific for breast cancer cell surface receptors, as any other variety of targets, is a balance between the affinity to the target and its frequency on the library pool. Therefore, the library heterogeneity is a critical step for the success of panning experiments. In this study, we initially used a commercial 12-mer library aiming to isolate highly specific peptides directed against potential biomarkers present on the cell surface of MDA-MB-231 cells. For this purpose, we used the conventional phage display methodology. The phage pool of the last round of phage display was subjected to preliminary assays against several cell lines (MDA-MB-231, MCF-10-2A, SK-BR-3, Hs 578 T and MDA-MB-435) using flow cytometry to evaluate its specificity for MDA-MB-231 cells. The flow cytometry results, presented in Additional file 4: Figure S1, clearly indicate the selectivity of the phage pool towards MDA-MB-231 cells, with statistical significance as compared to the remaining cell lines evaluated. Then, this preliminary analysis was refined to study the interaction of the phage pool with the MDA-MB-231 cells by immunohistochemistry. Additional file 5: Figure S2 demonstrates binding of the phage pool to MDA-MB-231 tissue sections (identified by green fluorescence in Additional file 5: Figure S2B), in contrast to the wild type M13KE phages, which exhibit no staining (Additional file 5: Figure S2A), thus clearly suggesting the capacity of the peptides selected by phage display techniques to interact with the target cells.

With these initial results we could confirm the possibility of obtaining specific and selective peptides for the MDA-MB-231 cell line and so, we enlarged the study using an additional phage display library, containing only 7 amino acids (7-mer library), as well as a more recently developed phage display methodology, Biopanning and rapid analysis of selective interactive ligands (BRASIL). A library of smaller peptides may offer an advantage over the 12-mer library on the strength of binding of the peptides selected. Additionally, BRASIL presents the advantages of being faster and using counter-selection (to remove peptides that bind to targets present on other cells), but can be limited by the use of suspended cells, potentially hiding surface receptors only present in the adherent state.

For each phage display methodology and library, eight rounds of panning were performed and the peptides obtained from the last panning round of each experimental set (details provided in Additional file 1: Table S1) are presented in Table 1. Also, the consensus sequence with the respective overall percentage was determined (Table 1).

**Table 1:** Conventional and modified BRASIL enrichment results based on binding affinity to the breast cancer MDA-MB-231 cell line and a counter selection with the non-tumorigenic MCF-10-2A cell line with the 7-mer and 12-mer libraries

Method	7-mer library		12-mer library	
	Phage clone	Sequence	Phage clone	Sequence
Conventional	3.1 (5/27)	PRLNTSP	6.2 (1/17)	TTFNSFGRVAIE
	3.1 (6/27)	PTLDVAP	6.2 (2/17)	TTFCSFGRVRIE
	3.1 (8/27)	PQLNVSP	6.2 (3/17)	TTFNSFGRVRIE
	3.1 (9/27)	PGAQVSP	6.2 (4/17)	TTFNSFGRVHWE
	3.1 (10/27)	PRTNVAP	6.2 (5/17)	TTFNSFGKVRIE
	3.1 (11/27)	PRKTVSP	6.2 (7/17)	TTYNSFGRVRIE
	3.1 (12/27)	PAMNVSP	6.2 (8/17)	TTEYSFGRTSTL
	3.1 (13/27)	PQENASP	6.2 (9/17)	DTFNSFGRVRIE
	3.1 (19/27)	PSLNVSP	6.2 (10/17)	TTFNSFGRVRIQ
	3.1 (20/27)	ARLNVAP	6.2 (12/17)	TTFNSFGRVRIE
	3.1 (21/27)	TMLMVRP	6.2 (13/17)	TNFNDFKRVRGE
	3.1 (22/27)	ARLNTQP	6.2 (14/17)	TTFSSFGRVRIG
	3.1 (23/27)	PMMAVAP	6.2 (15/17)	TTFNSNGRVWIE
	3.1 (26/27)	PRLNVSP	6.2 (16/17)	TTFNSFGRVRGG
	3.1 (27/27)	PRLNVSP	6.2 (17/17)	TTFNSFYRVRIE
	*Consensus (overall %)*	**PRLNVSP (70%)**	*Consensus (overall %)*	**TTFNSFGRVRIE (86%)**
BRASIL	1.3 (2/52)	PRQNQSP	5.3 (1/45)	WWFNSFGRVRIE
	1.3 (3/52)	QLKNVTP	5.3 (3/45)	WAFNSFGRVRIE
	1.3 (5/52)	PRLNVAT	5.3 (5/45)	WWFNKFGKVRIE
	1.3 (6/52)	PRLNVTT	5.3 (14/45)	WWFNSFGRVRIE
	1.3 (7/52)	PRWAVSP	5.3 (16/45)	IWFNSFFRVRIE
	1.3 (8/52)	PRLNHSP	5.3 (19/45)	WWFFSFGRVRIE
	1.3 (10/52)	PQMTAMP	5.3 (20/45)	GWFNSFGRWSWL
	1.3 (11/52)	MFLNGAP	5.3 (23/45)	WWFNSFGRVRIE
	1.3 (15/52)	TRLQVSP	5.3 (43/45)	WWFFSFGRWSWL
	1.3 (19/52)	WRMAHSP	5.3 (45/45)	WWFNSFGRVRIE
	*Consensus (overall %)*	**PRLNVSP (60%)**	*Consensus (overall %)*	**WWFNSFGRVRIE (87%)**

Bold words represent the consensus (overall %) for the two methods

Conventional phage display and BRASIL methodologies resulted in similar consensus sequences. In fact, for the 7-mer library the sequence is identical (PRLNVSP), and for the 12-mer library only the first two amino acids are different (TTFNSFGRVRIE for the conventional method and WWFNSFGRVRIE for BRASIL). On the other hand, comparing the two libraries herein used, the consensus peptides obtained are very different. Furthermore, the overall percentage of consensus is higher for the commercial 12-mer library (86 %, 87%) than for the home-made 7-mer library (70 %, 60%).

### Binding assays

**Fig. 1:**
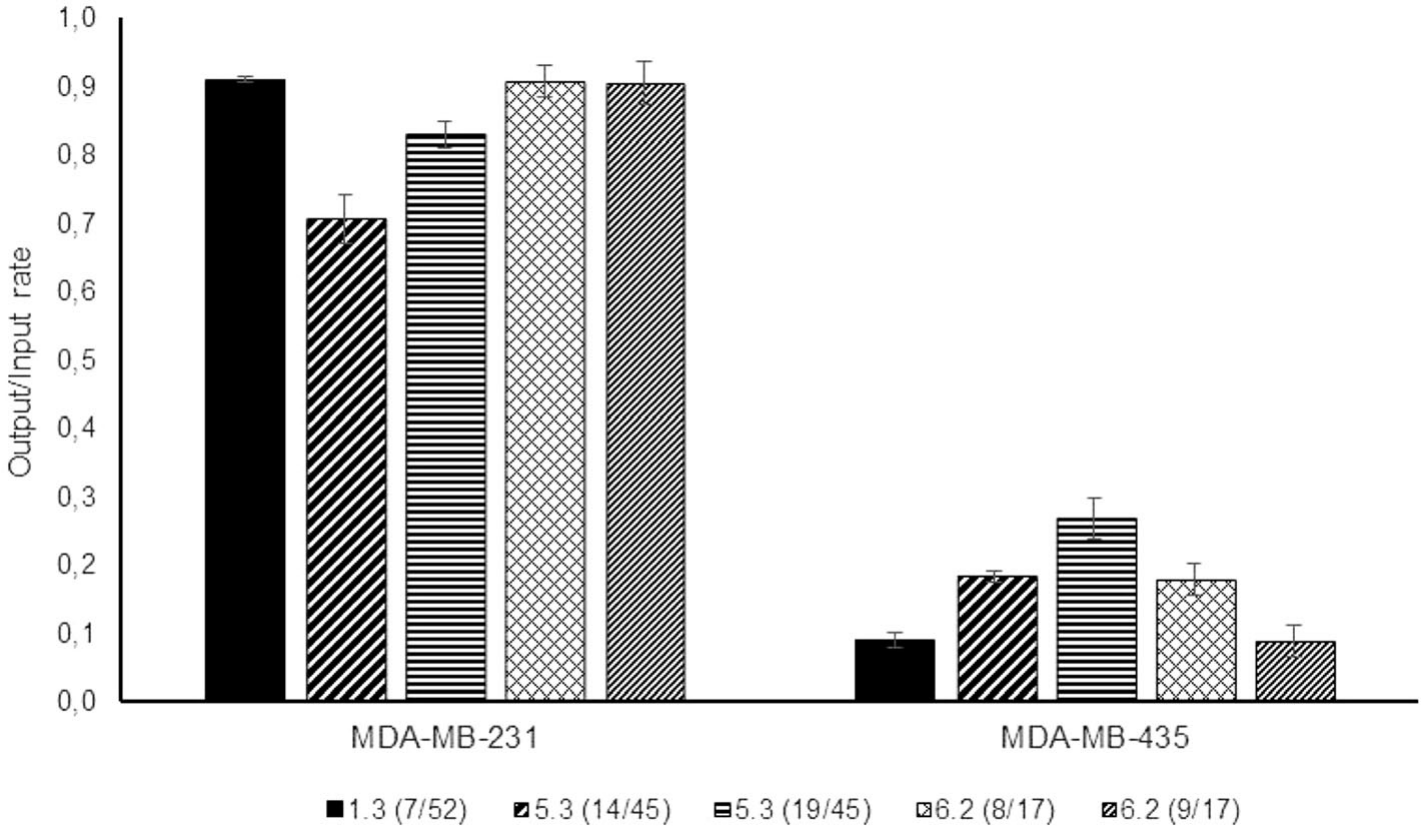
Binding assays of selected peptides against MDA-MB-231 and MDA-MB-435 cell lines, using an initial phage concentration of 1×10^11^ PFUs.mL^.1^. Results are expressed as the ratio between the concentration of phages bound to the cells (output) and the initial phage concentration used (input)

Peptides 1.3(7/52) (PRWAVSP), 5.3(14/45) (WWFNSFGRVRIE), 5.3(19/45) (WWFFSFGRVRIE), 6.2(8/17) (TTEYSFGRTSTL) and 6.2(9/17) (DTFNSFGRVRIE) were selected among those identified by phage display to assess in vitro for their binding affinity to MDA-MB-231 (claudin-low breast cancer subtype), by incubation of the cells with M13KE phages containing each peptide in analysis. The melanoma MDA-MB-435 cells were used as a negative control to evaluate the specificity of the peptides for the breast cancer MDA-MB-231 cells. The results, presented as the ratio between the concentration of phages bound to each cell line and the initial phage concentration used, are shown in Fig. 1.

The phages displaying the selected peptides have a higher binding affinity to MDA-MB-231 cells than to MDA-MB-435 cells, with the differences ranging from 0.55 (corresponding to 6 logs, for peptides 5.3(14/45), sequence WWFNSFGRVRIE and 5.3(19/45), sequence WWFFSFGRVRIE) to 0.80 (9 logs, for peptides 1.3(7/52), sequence PRWAVSP and peptide 6.2 (9/17) sequence DTFNSFGRVRIE), with the latter two demonstrating the most promising results in terms of specificity and binding strength.

**Fig. 2:**
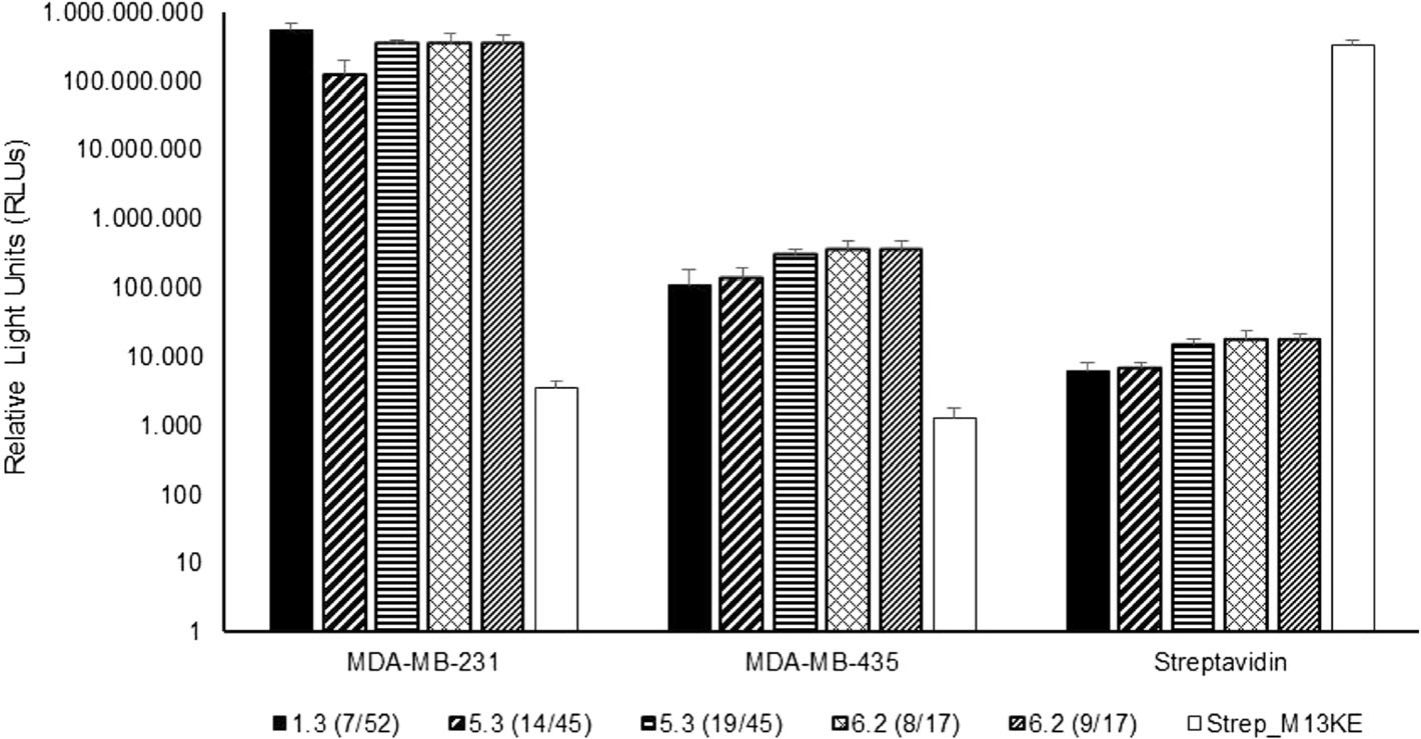
Relative light units (RLUs) obtained for the selected peptides assessed by ELISA against MDA-MB-213 and MDA-MB-435 cell lines, as well as against the control streptavidin, according to the New England BioLabs phage display manual

Enzyme-linked immunosorbent assays (ELISA) were performed with the selected peptides against MDA-MB-231 cells. MDA-MB-435 cells were used as a negative control and streptavidin as a positive control (using an M13KE phage displaying affinity peptides towards streptavidin). Results were read in a luminometer and the relative light units (RLUs) obtained are shown in Fig. 2.

The ELISA assays are in good agreement with the results obtained from the binding assays described above (Fig. 1), with all peptides showing higher affinity to the MDA-MB-231 cells than to the MDA-MB-435 cells. The differences observed between the two cell lines range from 3 to 4 logs.

### Library analysis

The peptides obtained in this work were compared to previously reported peptides (specific to breast cancer cells) to assess possible similarities. This was performed using pair-wise sequence alignments to prevent the bias towards the discovery of consensus sequence obtained when using multiple sequence alignments. Blosum45, 50 and 62 were used and compared, with the Blosum45 matrix being chosen to score the alignments since it is more adequate to score divergent sequences. An initial analysis demonstrated a high impact of sequence length on the similarity computation (see Additional file 6: Figure S3). Therefore, to consider the local background of each sequence regarding the alignment score, the CLR algorithm was adapted to this context. This algorithm has been used successfully to take the local background into account when assessing similarities between gene expression profiles or metabolite concentrations [27, 28].

**Fig. 3:**
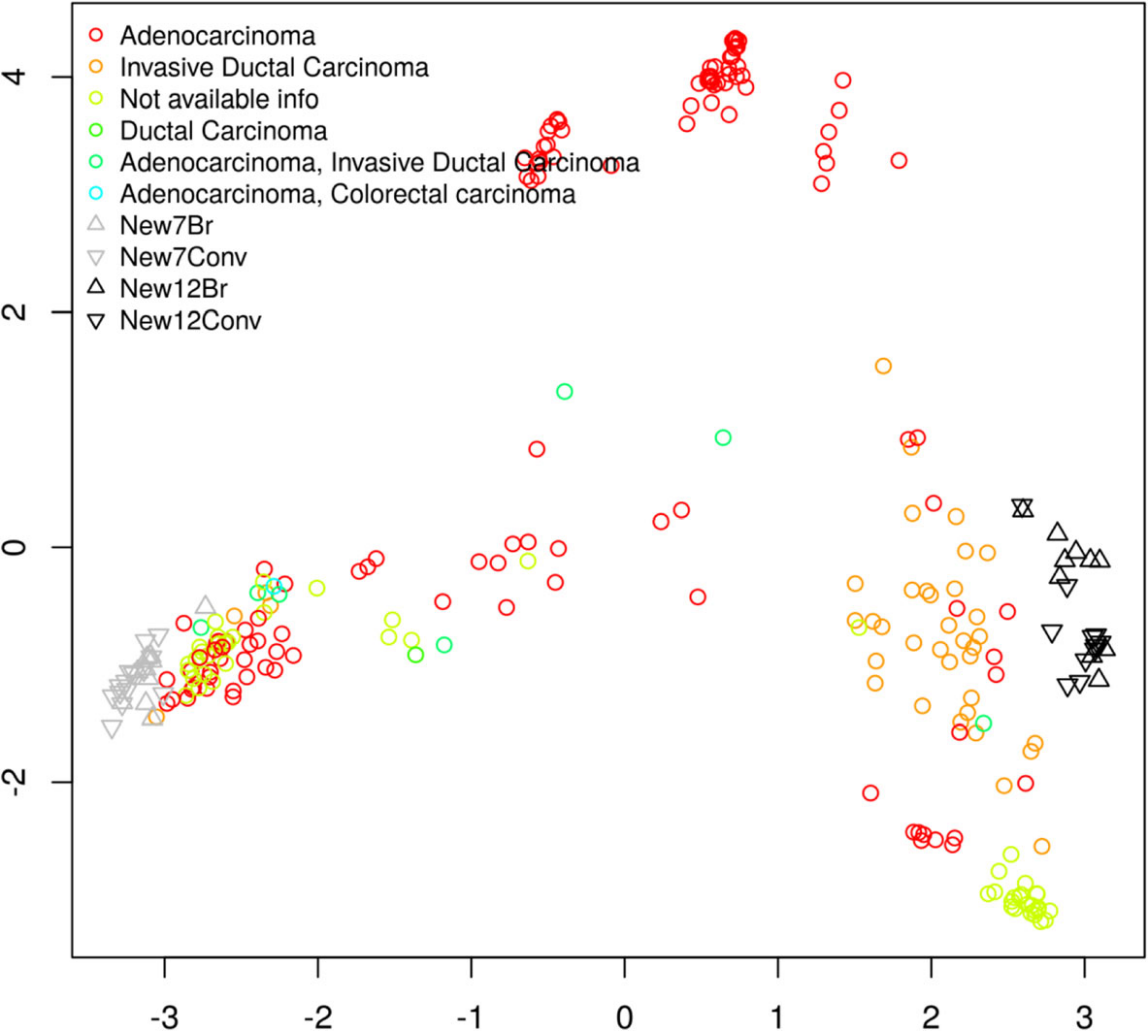
Multidimensional scaling of the peptides identified in this work against MDA-MB-231 cells and previously reported peptides against breast cancer cells. New7Br: 7-mer peptides obtained in this work using the BRASIL methodology; New7Conv: 7-mer peptides obtained in this work using the conventional methodology; New12Br: 12-mer peptides obtained in this work using the BRASIL methodology; New12Conv: 12-mer peptides obtained in this work using the conventional methodology; remaining peptides are grouped according to the breast cancer type targeted (adenocarcinoma, invasive ductal carcinoma, ductal carcinoma, adenocarcinoma and invasive ductal carcinoma, adenocarcinoma and colorectal carcinoma, and peptides with no available information)

The multidimensional scaling of the peptides can be seen in Fig. 3, where graphical distances between the item represents the (dis)similarities between the sequences. The algorithm places the newly identified sequences in the outskirts of the figure, indicating an average low similarity shared with previously identified peptides.

**Fig. 4:**
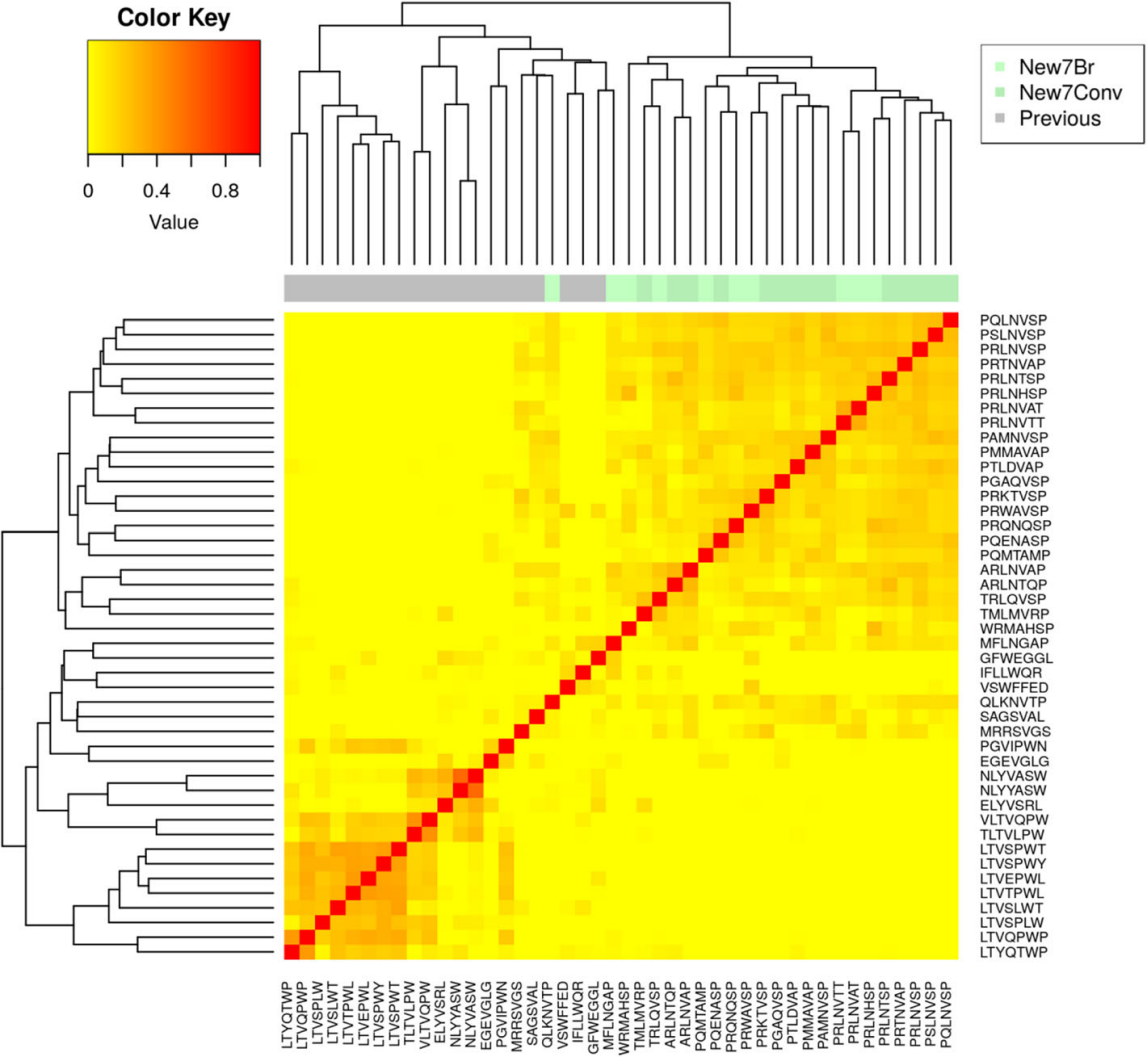
Heatmap representation of the similarities between the 7-mer peptides identified in this work with those previously reported. New7Br: 7-mer peptides obtained in this work using the BRASIL methodology; New7Conv: 7-mer peptides obtained in this work using the conventional methodology; Previous: 7-mer peptides reported in previous studies

**Fig. 5:**
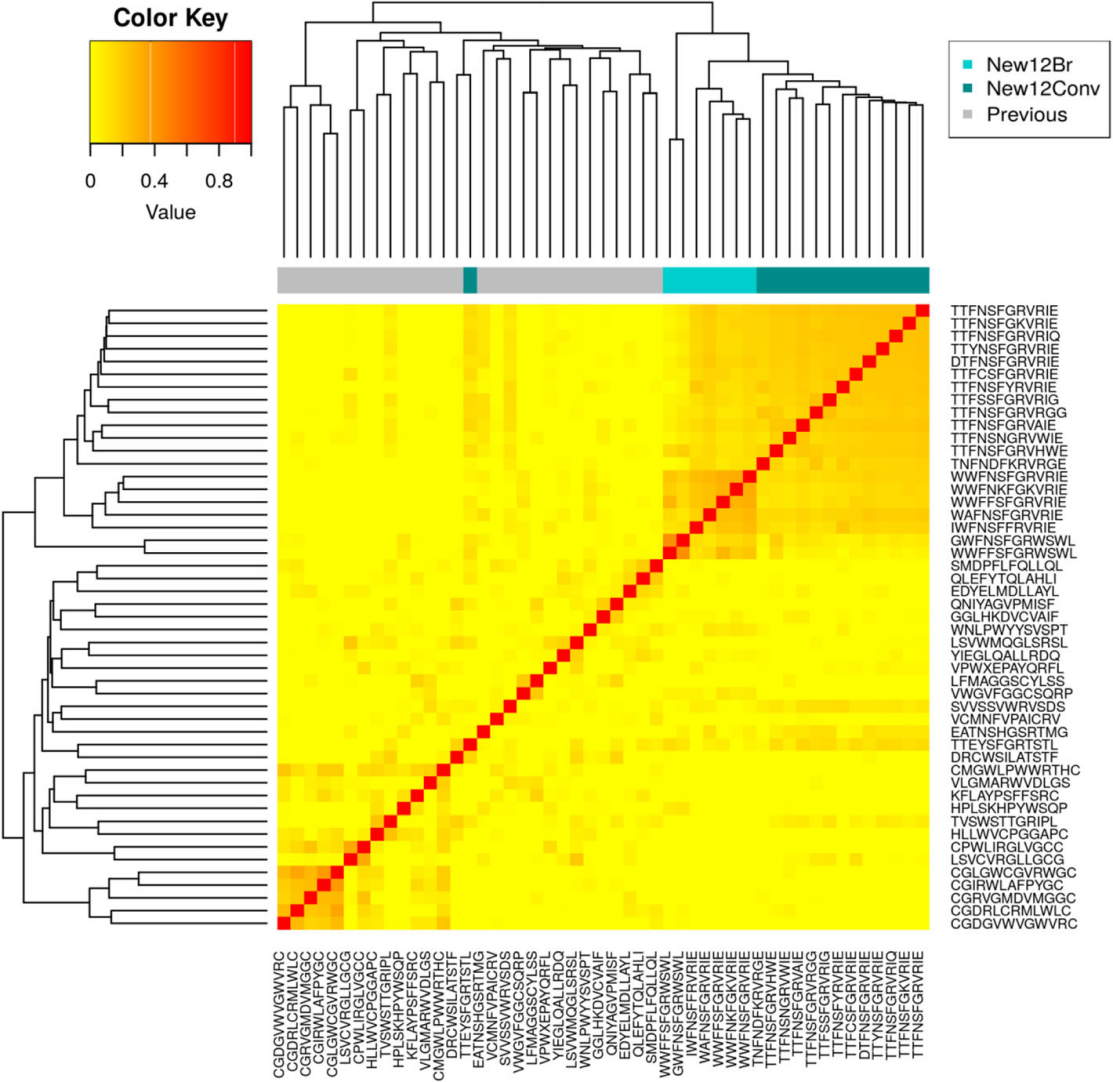
Heatmap representation of the similarities between the 12-mer peptides identified in this work with those previously reported. New12Br: 12-mer peptides obtained in this work using the BRASIL methodology; New12Conv: 12-mer peptides obtained in this work using the conventional methodology; Previous: 12-mer peptides reported in previous studies

The similarities between all sequences were illustrated in a heatmap (Additional file 6: Figure S3). Even though the local context of each sequence has been considered, there was still a prevalence of association between sequences of similar length. Therefore, to fully consider the effect of this bias, separated heatmaps for the 7-mer (Fig. 4) and 12-mer peptides (Fig. 5) were built only considering peptides of the same length. Results show that indeed the newly identified peptides are far (in sequence space) from those previously reported.

### Docking studies

**Fig. 6:**
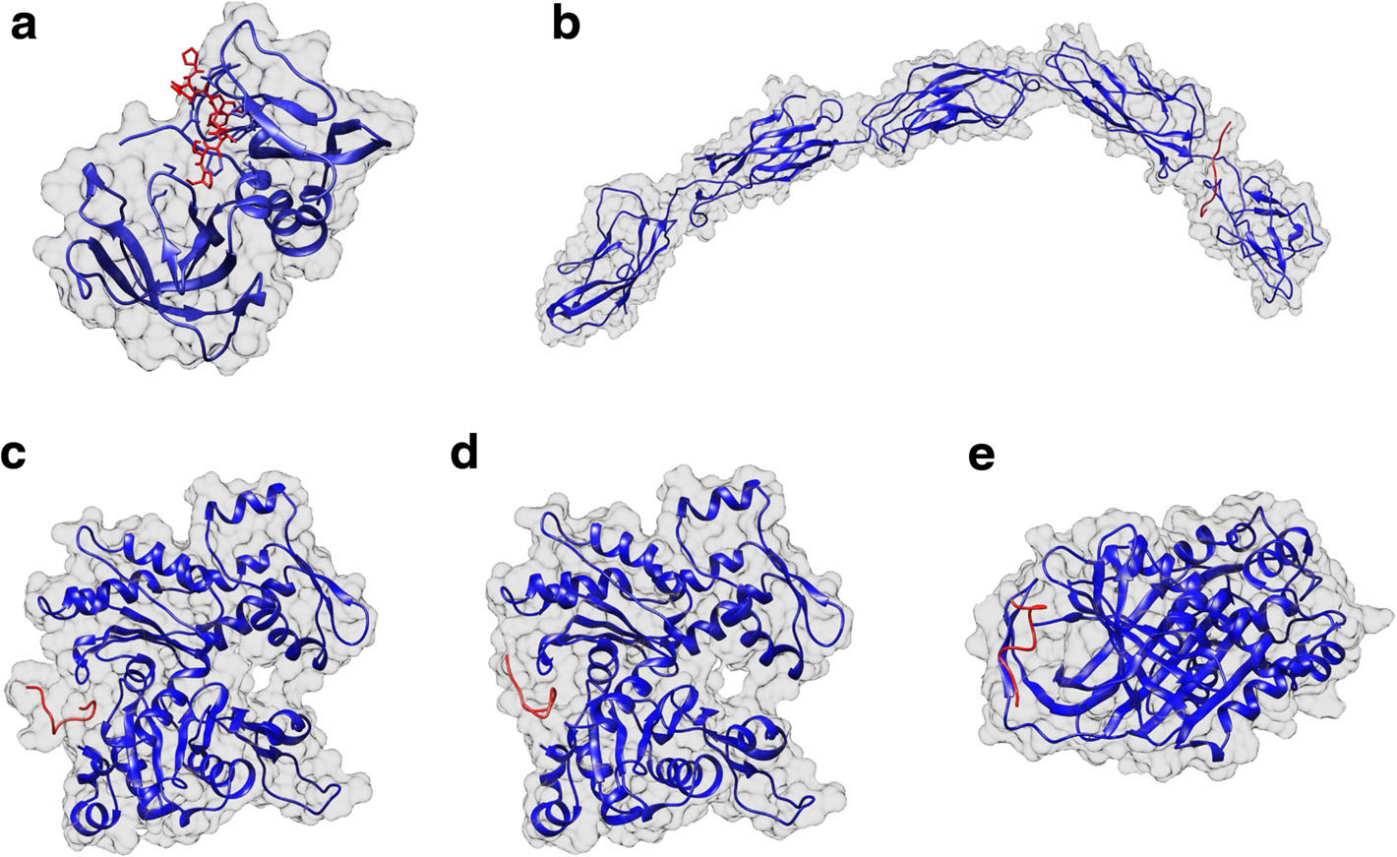
Tri-dimensional view of the peptides (*red*) docked to the respective biomarker (*blue*). **a** Peptide 1.3 (7/52) docked to Metalloproteinase Inhibitor 1, **b** peptide 5.3 (14/45) docked to E- cadherin, **c** peptide 5.3 (19/45) docked to β-Actin. **d** peptide 6.2 (8/17) docked to β-Actin, and **e** peptide 6.2 (9/17) docked to Plasminogen activator inhibitor 1. Images were obtained with Chimera version 1.10.2

A structural bioinformatics approach was implemented to identify potential targets of the peptides in the MDA-MB-231 cells. For this purpose, established biomarkers present in breast cancer cells were retrieved from the literature using search engines such as PubMed (with keywords “breast cancer biomarkers”, “MDA-MB-231 biomarkers”, “breast cancer surface markers”, “MDA-MB-231 surface markers”, and from open source databases (e.g., SurfaceomeDB). The proteins (biomarkers) were challenged by rigid body docking with the peptides using ClusPro 2.0. The results of the best docking model are shown in Table 2 and the tri-dimensional representation can be seen in Fig. 6. Additional information about energy values for all biomarkers is given in Additional file 7: Table S2.

**Table 2:** Data from the best docking model of the phage-display peptides against breast cancer biomarkers: biomarker, type of interaction, number of cluster members and lowest-energy weighted score (*E*)

Phage clone	Best docking model			
	Breast cancer biomarkers	Type of interaction ^a^	Cluster members ^b^	Lowest energy *E* ^c^
Conventional				
6.2 (8/17)	β-Actin	Hydrophobic-favoured	661	−1128.8
6.2 (9/17)	Plasminogen activator inhibitor 1	Hydrophobic-favoured	741	−1046.5
BRASIL				
1.3 (7/52)	Metalloprotease inhibitor 1	Hydrophobic-favoured	595	−1127
5.3 (14/45)	E-cadherin	Electrostatic-favoured	443	−1106
5.3 (19/45)	β-Actin	Hydrophobic-favoured	600	−1696.6

^a^ Coefficient weights of *E* formula are adapted for Balanced, Electrostatic-favored, Hydrophobic-favored or van der Waals and Electrostatic interactions

^b^ ClusPro 2.0 ranks models by cluster size. 1000 rotation/translation combinations of lowest score are chosen from 70,000 rotations performed, and are clustered together to find the ligand position with the most “neighbors” in 9 angstroms, becoming a cluster center and the neighbors the members of the cluster. A second cluster center is obtained with the remaining rotations and so on. So the most members on the cluster, the most significant the result

^c^ Weighted score is calculated according to formula *E* = 0.40*E*rep + −0.40*E*_att_ + 600*E*_elec_ + 1.00*E*_DARS_ (Balanced), *E* = 0.40*E*_rep_ + −0.40*E*_att_ + 1200*E*_elec_ + 1.00*E*_DARS_ (Electrostatic-favored), *E* = 0.40*E*_rep_ + −0.40*E*_att_ + 600*E*_elec_ + 2.00*E*_DARS_ (Hydrophobic-favored), or *E* = 0.40*E*_rep_ + −0.10*E*_att_ + 600*E*_elec_ + 0.00*E*_DARS_ (Van der Waals and Electrostatic)

Peptides 1.3 (7/52) (PRWAVSP) and 6.2 (9/17) (DTFNSFGRVRIE), which were found to have the best selective binding to MDA-MB-231, seem to interact with the biomarkers Metalloproteinase Inhibitor 1 (TIMP-1) and Plasminogen activator inhibitor 1 precursor (PAI1), respectively. The MDA-MB-231 biomarker β-actin, associated with breast cancer metastasis [37], is also targeted by two peptides, 5.3 (19/45) (WWFFSFGRVRIE) and 6.2 (8/17) (TTEYSFGRTSTL).

## Discussion

Claudin-low breast cancer subtype is characterized by an aggressive and highly metastatic nature that combined with the absence of known specific molecular biomarkers results in a very poor prognosis of therapeutic success [8, 9]. The identification of peptides that could specifically recognize this type of breast cancer may open new perspectives for the development of targeted therapies leading to improved prognosis. Herein, we applied a phage display methodology coupled with bioinformatics analysis to identify a peptide specific for a cell line representing the claudin-low breast carcinoma, namely the MDA-MB-231 cell line.

In a first stage, a conventional panning methodology and a commercial 12-mer M13KE library were used to identify a specific peptide against the MDA-MD-231 cell line. The phage pool obtained in the last round of selection was firstly evaluated by flow cytometry for specificity and selectivity against MDA-MB-231 cells, as well as cell lines from other important cancer subtypes MCF-10-2A, SK-BR-3 and Hs 578 T) and the melanoma MDA-MB-435 cell line (Additional file 4: Figure S1). The results indicate a strongest affinity for the target cells, but also a good binding to the MCF-10-2A and SK-BR-3 cell lines. The lowest affinity was detected for the MDA-MB-435 cells, which was expected since they were used in counter-selection. Afterwards, immunohistochemistry analysis of the phage pool against tissue sections of the target cells (MDA-MB-231) was carried out (Additional file 5: Figure S2), demonstrating the binding affinity of the pool for the target. These initial results proved the feasibility of obtaining a specific peptide targeting the MDA-MB-231 cells using phage display approaches. To increase the possibility of identifying peptides with strong binding affinities, an additional phage display methodology (BRASIL) and a home-made 7-mer M13KE library were included in this study.

The 7-mer and 12-mer libraries led to different consensus sequences (Table 1). This indicates a strong influence of the library on the phage display results, which was expected due to the difference in length of the peptides (7 or 12 amino acids). Indeed, although the 7-mer library is adequate for a biopanning strategy, it is more useful for targets requiring binding elements concentrated in a short sequence of amino acids [38, 39]. In turn, the 12-mer library may have an advantage if the binding amino acids (which most of the times are less than 12) are spread out over the peptide sequence [38, 40]. Moreover, the 12-mer library may also increase the effective peptide diversity since each 12-mer peptide contains 7-mer peptides with different flanking sequences [39]. However, due to the increased length of the 12-mer peptides, it is possible that sequences with multiple weak binding are selected instead of sequences with few strong bindings [38]. Nevertheless, libraries of both lengths have been successfully used for biopanning experiments, as also observed herein.

Comparing the phage display methodologies, for the 7-mer library, both BRASIL and conventional phage display resulted in the same consensus sequence. However, for the 12-mer library the consensus sequence differed between the two methodologies in the first two amino acids, perhaps because as explained above, these two amino acids may not be relevant for the strength of binding of the 12-mer peptide. However, these methods - conventional panning and BRASIL - do not display a significant difference. Since BRASIL is simple and faster, it is possible to say that this method is preferable for practical purposes [21].

Five peptides from the phage pools were selected to evaluate their binding affinities through different experimental assays. Both phage forming units (PFUs, Fig. 1) and ELISA (Fig. 2) assays suggest that all peptides exhibit specificity to the MDA-MB-231 cells with a lower binding capacity to MDA-MB-435 cells, as expected since this latter cell line was used in the counter-selection. These differences were of up to 80% (corresponding to 9 logs) in the PFUs assays (Fig. 1) and 4 logs in the ELISA assays (Fig. 2). These differences of peptide affinity between target and non-target cells are in accordance with values previously reported for phage display studies identifying peptides specific for other cancer cells, e.g., renal carcinoma A498 cells [41], breast cancer SKBR3 cells [42] and ovarian cancer HO8910 cells [43]. Among the peptides, 1.3(7/52) (PRWAVSP) and 6.2(9/17) (DTFNSFGRVRIE) present the most promising results for targeted therapies of claudin-low cancer subtype due to the higher binding strengths and selectivity for the MDA-MB-231 cells.

Bioinformatics analysis (Table 2) indicate that peptides 1.3(7/52) (PRWAVSP) and 6.2(9/17) (DTFNSFGRVRIE) specifically target TIMP-1 and PAI1, respectively, with both biomarkers being related to breast cancer and to MDA-MB-231 cells. TIMP-1 is often overexpressed in many malignancies and is associated with increased histological grade, lymph-node and distant metastasis and decreased survival in breast cancer [44]. It is present and overexpressed in the MDA-MB-231 cells but also in Hs 578 T cells, with no expression on the remaining cell lines evaluated [45]. Although TIMP-1 has been considered a potential target for prognosis and therapeutic purposes, to our knowledge no specific peptide, antibody, aptamer or other molecule has been identified against this protein. Hence, the peptide herein identified represents a promising development for the establishment of prognosis tools, as well as targeted therapies. On the other hand, PAI1 is considered a prognostic marker due to a strong correlation with tumor aggressiveness and poor clinical outcome in breast cancer [46]. It is highly expressed in MDA-MB-231 cells, but also on MCF-10-2A and SK-BR-3 cells, exhibiting low levels of expression on Hs 578 T and MDA-MB-435 cells, which is somewhat in accordance with the preliminary flow cytometry results (Additional file 4: Figure S1) obtained for the phage pool from which the peptide 6.2(9/17) was obtained. Several aptamers have been developed that bind and inhibit PAI1, exhibiting potential therapeutic applications as anti-metastatic agents [47, 48]. The peptide here identified represents another alternative also with therapeutic and prognosis potential.

From the bioinformatics analysis, it is also interesting to note that peptides with different sequences (5.3 (19/45) sequence WWFFSFGRVRIE, and 6.2 (8/17) sequence TTEYSFGRTSTL) can exhibit affinities towards the same target (in this case the MDA-MB-231 biomarker β-actin, associated with breast cancer metastasis [37]), while similar peptides differing only in one amino acid have a different target (e.g., 5.3 (14/45), sequence WWFNSFGRVRIE targeting E- cadherin)). This indicates that not only the amino acid sequence, but also the tri-dimensional conformation of the peptides influence the peptide interactions with the cells.

Finally, the peptide 5.3 (14/45) (WWFNSFGRVRIE) exhibited the highest affinity for a biomarker that is not present in the MDA-MB-231 cells (E- cadherin) [49], although showing a similar affinity for a MDA-MB-231 biomarker (α-1-antichymotrypsin) (see Additional file 7: Table S2). This might explain the lowest selective affinity of this peptide for MDA-MB-231 cells as demonstrated in the binding studies.

All the peptides identified in this study were compared with reported breast cancer specific peptides and no similarities have been found (Figs. 3, 4, and 5), thus strongly supporting their novelty.

## Conclusions

In this work we identified new peptides specific for the MDA-MB-231 cells, which is representative of the claudin-low subtype of breast carcinomas, using phage display aided by bioinformatics tools. The methodology used together with the interpretation of phage display results (peptide sequences) being aided by bioinformatics approaches can be very useful to predict the potential cell targets (biomarkers) and to isolate peptides that are specific for the desired cells from those binding to other cancer subtypes. The selected peptides, PRWAVSP and DTFNSFGRVRIE, exhibit a strong binding to the MDA-MB-231 cells and a good specificity as demonstrated by the low binding to the MDA-MB-435 cells. Such peptides can be a valuable contribute towards future clinical applications through the development of more specific and targeted therapeutic solutions against the claudin-low breast cancer subtype.

## Additional files

Additional file 1: Table S1. Experimental setting of the phage display experiments, with BRASIL (B) and Conventional (C) methodologies, using the 7-mer and the 12-mer libraries. (DOCX 27 kb)
Additional file 2: Table S3. Breast cancer specific peptides reported in the literature, with amino acid sequence, sequence size, breast cancer stage, cell line targeted, cancer histological subtype and PubMed unique identifier number (PMID). (DOCX 58 kb)
Additional file 3: Table S4. Data collected for potential biomarkers of breast cancer cells, retrieved from Kyoto Encyclopedia of Genes and Genomes (KEGG), Uniprot, GenBank, and Protein Data Bank (PDB), with those present in MDA-MB-231 represented in bold. (DOCX 27 kb)
Additional file 4: Figure S1. Flow cytometry results, in terms of percentage of binding, of the phage pool from the last round of 12-mer conventional panning against normal breast cell line MCF-10-2A, breast cancer cell lines MDA-MB-231, SK-BR-3, Hs 578 T and MDA-MB-435 cell line [19]. Statistically significant (*P*) differences are represented by ***. (DOCX 43 kb)
Additional file 5: Figure S2. Immunofluorescence staining of MDA-MB-231 tissue sections. Sections were incubated with (A) wild-type M13KE phage particles and (B) M13KE phage particles of the phage pool from the last round of conventional phage display. Images were acquired with blue filter (1), green filter (2) and filter overlapping (3). Peptide affinity was detected using a primary anti-M13 antibody and a secondary goat anti-rabbit FITC conjugate antibody. Images were acquired using an inverted LEICA DMI 3000B (Leica Mycrosystems) with incorporated camera (Model DFC 450C). Scale bar of 10 μm. (DOCX 1254 kb)
Additional file 6: Figure S3. Heatmap representation of the similarities between all peptides identified in this work with those previously reported. New12Br: 12-mer peptides obtained in this work using the BRASIL methodology; New12Conv: 12-mer peptides obtained in this work using the conventional methodology; Previous: 12-mer peptides reported in previous studies. Legend bar on the right represents the peptides of x-mer in different colours. (DOCX 398 kb)
Additional file 7: Table S2. Data from docking analysis with the selected 7-mer and 12-mer phage display peptides against breast cancer biomarkers retrieved from the literature: lowest energy weighted score (*E*), cluster members (CM) and type of interaction (I). Biomarkers present in MDA-MB-231 cells and best model scores are given in bold. (DOCX 41 kb)

## Abbreviations

BRASIL: Biopanning and rapid analysis of selective interactive ligands
BSA: Bovine serum albumin
CLR: Context likelihood of relatedness
DAPI: 4′, 6 - diamidino-2-phenylindole
DMEM: Dulbecco’s Modified Eagle Medium
EDTA: Ethylenediaminetetraacetic acid
ELISA: Enzyme-linked immunosorbent assay
ER: Estrogen receptor
FBS: Fetal bovine serum
FITC: Fluorescein isothiocyanate
HER2: Human epidermal growth factor 2 receptor
IPATIMUP: Institute of Molecular Pathology and Immunology at the University of Porto
KEGG: Kyoto Encyclopedia of Genes and Genomes
PAI1: Plasminogen activator inhibitor 1 precursor
PBS: Phosphate buffered-saline
PBST: Phosphate buffered-saline with Tween-20
PDB: Protein data bank
PEG: Polyethylene glycol
PFU: Plaque forming unit
PR: Progesterone receptor
RLU: Relative light unit
ssDNA: Single-stranded DNA
TBS: Tris buffered saline
TBST: Tris buffered saline with Tween-20
TE: Tris-EDTA
TIMP-1: Metalloproteinase inhibitor 1
