# Supplementary material for: Screening and characterization of novel specific peptides targeting MDA-MB-231 claudin-low breast carcinoma by computer-aided phage display methodologies
Source: BMC Cancer. 2016 Nov 14;16:881. doi: 10.1186/s12885-016-2937-2 (PMC5109716; doi:10.1186/s12885-016-2937-2)
Supplement: Additional file 1: Table S1. — Experimental setting of the phage display experiments, with BRASIL (B) and Conventional (C) methodologies, using the 7-mer and the 12-mer libraries. (DOCX 27 kb) [file 12885_2016_2937_MOESM1_ESM.docx]

Additional file 1: **Table S1.** Experimental setting of the phage display experiments, with BRASIL (B) and Conventional (C) methodologies, using the 7-mer and the 12-mer libraries

| **CODE #** | **Library** | **Methodology** | **Targeting** | **Biopanning^a^** | **CS^b^** | **Total PFUs (after panning)^c^** | **Fold reduction^d^** |
| --- | --- | --- | --- | --- | --- | --- | --- |
| 1.1 | 7-mer | B | MDA-MB-231 | P1 | Y | 2.0x10^11^ | N/A |
| 1.2 | 7-mer | B | MDA-MB-231 | P2 | Y | 1.2x10^8^ | 6.0x10^-4^ |
| 1.3 | 7-mer | B | MDA-MB-231 | P8 | Y | 5.2x10^2^ | 2.6x10^-9^ |
| 2.1 | 7-mer | C | MDA-MB-231 | P1 | N | 2.0x10^11^ | N/A |
| 2.2 | 7-mer | C | MDA-MB-231 | P3 | N | 2.1x10^8^ | 1.1x10^-3^ |
| 2.3 | 7-mer | C | MDA-MB-231 | P8 | N | 1.7x10^1^ | 8.5x10^-11^ |
| 3.1 | 7-mer | C | MDA-MB-231 | P8 | Y | 2.7x10^1^ | 1.4x10^-10^ |
| 4.1 | 7-mer | C | Streptavidin (control) | P1 | N | 2.0x10^11^ | N/A |
| 4.2 | 7-mer | C | Streptavidin (control) | P2 | N | 5.6x10^10^ | 2.8x10^-1^ |
| 4.3 | 7-mer | C | Streptavidin (control) | P3 | N | 3.5x10^9^ | 1.8x10^-2^ |
|  |  |  |  |  |  |  |  |
| 5.1 | 12-mer | B | MDA-MB-231 | P1 | Y | 2.0x10^11^ | N/A |
| 5.2 | 12-mer | B | MDA-MB-231 | P2 | Y | 5.6x10^10^ | 2.8x10^-1^ |
| 5.3 | 12-mer | B | MDA-MB-231 | P8 | Y | 3.2x10^3^ | 1.6x10^-8^ |
| 6.1 | 12-mer | C | MDA-MB-231 | P1 | N | 2.0x10^11^ | N/A |
| 6.2 | 12-mer | C | MDA-MB-231 | P8 | N | 1.2x10^1^ | 6.0x10^-11^ |
| 7.1 | 12-mer | C | Streptavidin (control) | P1 | N | 2.0x10^11^ | N/A |
| 7.2 | 12-mer | C | Streptavidin (control) | P2 | N | 4.9x10^10^ | 2.5x10^-1^ |
| 7.3 | 12-mer | C | Streptavidin (control) | P3 | N | 4.5x10^09^ | 2.3x10^-2^ |

^a^ P# – Panning round number #

^b^ Counter Selection

^c^ Phage titer at the end of each panning round.

^d^ Reduction of phage titer in each panning round. N/A means no fold reduction.
