# Supplementary material for: Screening and characterization of novel specific peptides targeting MDA-MB-231 claudin-low breast carcinoma by computer-aided phage display methodologies
Source: BMC Cancer. 2016 Nov 14;16:881. doi: 10.1186/s12885-016-2937-2 (PMC5109716; doi:10.1186/s12885-016-2937-2)
Supplement: Additional file 2: Table S3. — Breast cancer specific peptides reported in the literature, with amino acid sequence, sequence size, breast cancer stage, cell line targeted, cancer histological subtype and PubMed unique identifier number (PMID). (DOCX 58 kb) [file 12885_2016_2937_MOESM2_ESM.docx]

Additional file 2: **Table S3.** Breast cancer specific peptides reported in the literature, with amino acid sequence, sequence size, breast cancer stage, cell line targeted, cancer histological subtype and PubMed unique identifier number (PMID)

| **Peptide Sequence** | **Size** | **Stage** | **Cell Line** | **Histological subtype** | **PMID** |
| --- | --- | --- | --- | --- | --- |
| EGEVGLG | 7 | IV | MDA-MB-231, MCF-7 | Adenocarcinoma | 19825959 |
| MRRSVGS | 7 | IV | MDA-MB-231, MCF-7 | Adenocarcinoma | 19825959 |
| SSAVL | 5 | IV | MDA-MB-231, MCF-7 | Adenocarcinoma | 19825959 |
| VLI | 3 | IV | MDA-MB-231 | Adenocarcinoma | 19825959 |
| SAGSVAL | 7 | IV | MDA-MB-231 | Adenocarcinoma | 19825959 |
| FGVR | 4 | IV | MDA-MB-231, MCF-7 | Adenocarcinoma | 19825959 |
| GFWEGGL | 7 | IV | MDA-MB-231 | Adenocarcinoma | 19825959 |
| CGDGVWVGWVRC | 12 | N/A | BT-474 | Invasive Ductal Carcinoma | 20219829 |
| CGDRLCRMLWLC | 12 | N/A | BT-474 | Invasive Ductal Carcinoma | 20219829 |
| CGRVGMDVMGGC | 12 | N/A | BT-474 | Invasive Ductal Carcinoma | 20219829 |
| CGLGWCGVRWGC | 12 | N/A | BT-474 | Invasive Ductal Carcinoma | 20219829 |
| GGLHKDVCVAIF | 12 | N/A | BT-474 | Invasive Ductal Carcinoma | 20219829 |
| CMGWLPWWRTHC | 12 | N/A | BT-474 | Invasive Ductal Carcinoma | 20219829 |
| CPWLIRGLVGCC | 12 | N/A | BT-474 | Invasive Ductal Carcinoma | 20219829 |
| CGIRWLAFPYGC | 12 | N/A | BT-474 | Invasive Ductal Carcinoma | 20219829 |
| VCMNFVPAICRV | 12 | N/A | BT-474 | Invasive Ductal Carcinoma | 20219829 |
| KFLAYPSFFSRC | 12 | N/A | BT-474 | Invasive Ductal Carcinoma | 20219829 |
| LFMAGGSCYLSS | 12 | N/A | BT-474 | Invasive Ductal Carcinoma | 20219829 |
| DRCWSILATSTF | 12 | N/A | BT-474 | Invasive Ductal Carcinoma | 20219829 |
| HLLWVCPGGAPC | 12 | N/A | BT-474 | Invasive Ductal Carcinoma | 20219829 |
| VWGVFGGCSQRP | 12 | N/A | BT-474 | Invasive Ductal Carcinoma | 20219829 |
| LSVCVRGLLGCG | 12 | N/A | BT-474 | Invasive Ductal Carcinoma | 20219829 |
| LSVWMQGLSRSL | 12 | N/A | BT-474 | Invasive Ductal Carcinoma | 20219829 |
| VLGMARWVDLGS | 12 | N/A | BT-474 | Invasive Ductal Carcinoma | 20219829 |
| SVVSSVWRVSDS | 12 | N/A | BT-474 | Invasive Ductal Carcinoma | 20219829 |
| DMPGTVLP | 8 | IV | MCF-7 | Adenocarcinoma | 21050894 |

**Table S3.** Breast cancer specific peptides reported in the literature, with amino acid sequence, sequence size, breast cancer stage, cell line targeted, cancer histological subtype and PubMed unique identifier number (PMID) (continuation)

| **Peptide Sequence** | **Size** | **Stage** | **Cell Line** | **Histological subtype** | **PMID** |
| --- | --- | --- | --- | --- | --- |
| NRLKCRAQATHSAAPCIRGY | 20 | IV | SK-BR-3, T47D, BT474, MDA-MB-231, MDA-MB-361 | N/A | 16885375 |
| RQNSCTYSDARRWALCWSGE | 20 | IV | SK-BR-3, T47D, BT474, MDA-MB-231, MDA-MB-361 | N/A | 16885375 |
| QLNSCIHSGDRAIRGCMDWV | 20 | IV | SK-BR-3, T47D, BT474, MDA-MB-231, MDA-MB-361 | N/A | 16885375 |
| KYGLCRDETVFPSHSCTFTG | 20 | IV | SK-BR-3, T47D, BT474, MDA-MB-231, MDA-MB-361 | N/A | 16885375 |
| GSPQCPGGFNCPRCDCGAGY | 20 | IV | SK-BR-3, T47D, BT474, MDA-MB-231, MDA-MB-361 | N/A | 16885375 |
| GTGSCGYGKLHTGYWCSYFP | 20 | IV | SK-BR-3, T47D, BT474, MDA-MB-231, MDA-MB-361 | N/A | 16885375 |
| NSSSCDTSVVRSTWACILQP | 20 | IV | SK-BR-3, T47D, BT474, MDA-MB-231, MDA-MB-361 | N/A | 16885375 |
| VRAVCTTLKSRGHEECWSLQ | 20 | IV | SK-BR-3, T47D, BT474, MDA-MB-231, MDA-MB-361 | N/A | 16885375 |
| VYAQCGVNVRTGRGGCSRLM | 20 | IV | SK-BR-3, T47D, BT474, MDA-MB-231, MDA-MB-361 | N/A | 16885375 |
| VHMNCSWMRVSEGHPCESAD | 20 | IV | SK-BR-3, T47D, BT474, MDA-MB-231, MDA-MB-361 | N/A | 16885375 |
| GRQGCYEHLWRLIAWCAIFL | 20 | IV | SK-BR-3, T47D, BT474, MDA-MB-231, MDA-MB-361 | N/A | 16885375 |
| LRMTCAFGVAQRSADCALSS | 20 | IV | SK-BR-3, T47D, BT474, MDA-MB-231, MDA-MB-361 | N/A | 16885375 |
| SIVNCSAALTDLPTRCGGNI | 20 | IV | SK-BR-3, T47D, BT474, MDA-MB-231, MDA-MB-361 | N/A | 16885375 |
| CGTRCVRCQNGPEASCEQPL | 20 | IV | SK-BR-3, T47D, BT474, MDA-MB-231, MDA-MB-361 | N/A | 16885375 |
| TPLFCGNHGRQPSPLCMKWD | 20 | IV | SK-BR-3, T47D, BT474, MDA-MB-231, MDA-MB-361 | N/A | 16885375 |
| FTTVCRQPRGHEAIVCGSGK | 20 | IV | SK-BR-3, T47D, BT474, MDA-MB-231, MDA-MB-361 | N/A | 16885375 |
| APSFCGTAMLGASRYCYSGP | 20 | IV | SK-BR-3, T47D, BT474, MDA-MB-231, MDA-MB-361 | N/A | 16885375 |
| GARECESGGPGMRKLCTQIN | 20 | IV | SK-BR-3, T47D, BT474, MDA-MB-231, MDA-MB-361 | N/A | 16885375 |
| NNRACFRTSKGNPAECPYLG | 20 | IV | SK-BR-3, T47D, BT474, MDA-MB-231, MDA-MB-361 | N/A | 16885375 |
| GSLACQNIVVCVKKQCNALC | 20 | IV | SK-BR-3, T47D, BT474, MDA-MB-231, MDA-MB-361 | N/A | 16885375 |
| KRASCQNPLFSNFFVCGLSE | 20 | IV | SK-BR-3, T47D, BT474, MDA-MB-231, MDA-MB-361 | N/A | 16885375 |
| LPNFCMDTSGRAGPLCMGSE | 20 | IV | SK-BR-3, T47D, BT474, MDA-MB-231, MDA-MB-361 | N/A | 16885375 |
| RHTVCRVSLSSVQGSCSHEY | 20 | IV | SK-BR-3, T47D, BT474, MDA-MB-231, MDA-MB-361 | N/A | 16885375 |
| TLTVLPW | 7 | IV | SK-BR-3 | Adenocarcinoma | 12490548 |
| LTVEPWL | 7 | IV | SK-BR-3 | Adenocarcinoma | 12490548 |
| LTVSPWY | 7 | IV | SK-BR-3, T47D | Adenocarcinoma | 12490548 |
| LTVSPLW | 7 | IV | SK-BR-3 | Adenocarcinoma | 12490548 |
| LTVTPWL | 7 | IV | SK-BR-3 | Adenocarcinoma | 12490548 |

**Table S3.** Breast cancer specific peptides reported in the literature, with amino acid sequence, sequence size, breast cancer stage, cell line targeted, cancer histological subtype and PubMed unique identifier number (PMID) (continuation)

| **Peptide Sequence** | **Size** | **Stage** | **Cell Line** | **Histological subtype** | **PMID** |
| --- | --- | --- | --- | --- | --- |
| LTVQPWP | 7 | IV | SK-BR-3 | Adenocarcinoma | 12490548 |
| LTVSPWT | 7 | IV | SK-BR-3 | Adenocarcinoma | 12490548 |
| VLTVQPW | 7 | IV | SK-BR-3 | Adenocarcinoma | 12490548 |
| LTVSLWT | 7 | IV | SK-BR-3 | Adenocarcinoma | 12490548 |
| PGVIPWN | 7 | IV | SK-BR-3 | Adenocarcinoma | 12490548 |
| LTYQTWP | 7 | IV | SK-BR-3 | Adenocarcinoma | 12490548 |
| ELYVSRL | 7 | IV | SK-BR-3 | Adenocarcinoma | 12490548 |
| NLYVASW | 7 | IV | SK-BR-3 | Adenocarcinoma | 12490548 |
| WNLPWYYSVSPT | 12 | IV | SK-BR-3, T47D, MCF-7 | Adenocarcinoma | 12490548 |
| QNIYAGVPMISF | 12 | N/A | BT-483 | Invasive Ductal Carcinoma | 23776619 |
| EATNSHGSRTMG | 12 | N/A | BT-483 | Invasive Ductal Carcinoma | 23776619 |
| TVSWSTTGRIPL | 12 | N/A | BT-483 | Invasive Ductal Carcinoma | 23776619 |
| QLEFYTQLAHLI | 12 | N/A | BT-483 | Invasive Ductal Carcinoma | 23776619 |
| SMDPFLFQLLQL | 12 | N/A | BT-483 | Invasive Ductal Carcinoma | 23776619 |
| VSWFFE | 6 | IV | MCF-7 (ER + tumors) | Adenocarcinoma | 15468062 |
| VSWFFED | 7 | IV | MCF-7 (ER + tumors) | Adenocarcinoma | 15468062 |
| CPGPEGAGC | 9 | IV | *In vivo* (MMTV PyMT mice) | N/A | 11854520 |
| LPALDPTKRWFFETK | 15 | IV | MCF-7 | Adenocarcinoma | 11836009 |
| EMTPVNPG | 8 | IV | T-47D | Invasive Ductal Carcinoma | 10727847 |
| GCGGRGDGGC | 10 | IV | MDA-MB-231, MCF-7 | Adenocarcinoma | 16328042 |
| RGDWPC | 6 | IV | MCF-7 | Adenocarcinoma | 16619296 |
| FCFWKTCW | 8 | N/A | N/A | N/A | 1968467 |
| FCYWKVCT | 8 | N/A | N/A | N/A | 1968467 |
| FCYWKVCW | 8 | IV | ZR-75-1 | Ductal Carcinoma | 9179235 |
| FCFWKTCT | 8 | N/A | N/A | N/A | 1968467 |
| GGGQWAVGHLMNH | 13 | IV | T-47D | Invasive Ductal Carcinoma | 17532883 |
| ELYENVGMYC | 10 | IV | MDA-MB-453 | Adenocarcinoma | 14521914 |
| GRGDTP | 6 | IV | Hs 578T, T47D | Invasive Ductal Carcinoma | 10194517 |

**Table S3.** Breast cancer specific peptides reported in the literature, with amino acid sequence, sequence size, breast cancer stage, cell line targeted, cancer histological subtype and PubMed unique identifier number (PMID) (continuation)

| **Peptide Sequence** | **Size** | **Stage** | **Cell Line** | **Histological subtype** | **PMID** |
| --- | --- | --- | --- | --- | --- |
| YVPFP | 5 | IV | T47D | Invasive Ductal Carcinoma | 8920997 |
| YPFP | 4 | IV | T47D | Invasive Ductal Carcinoma | 7585646 |
| DKIFGSLAFL | 10 | IV | MCF-7, MDA-MB-231 and T47D | Adenocarcinoma, Invasive Ductal Carcinoma | 19414394 |
| DPDTRP | 6 | IV | MCF-7, MDA-MB-231 and T47D | Adenocarcinoma, Invasive Ductal Carcinoma | 19414394 |
| DKIFGSLAFLPDTRP | 15 | IV | MCF-7, MDA-MB-231 and T47D | Adenocarcinoma, Invasive Ductal Carcinoma | 19414394 |
| DEPPT | 5 | IV | MCF-7, MDA-MB-231 and T47D | Adenocarcinoma, Invasive Ductal Carcinoma | 19414394 |
| DEPPTPDTRP | 10 | IV | MCF-7, MDA-MB-231 and T47D | Adenocarcinoma, Invasive Ductal Carcinoma | 19414394 |
| EPPT | 4 | IV | MCF-7, MDA-MB-231 and T47D | Adenocarcinoma, Invasive Ductal Carcinoma | 19414394 |
| EDYELMDLLAYL | 12 | IV | MCF-7 | Adenocarcinoma | 17504878 |
| KGVSLSYR | 8 | N/A | MMTV-PyMT mouse | N/A | 20830712 |
| RTRYED | 6 | N/A | MMTV-PyMT mouse | N/A | 20460372 |
| GMMYRS | 6 | N/A | MMTV-PyMT mouse | N/A | 20460372 |
| RWRTNF | 6 | N/A | MMTV-PyMT mouse | N/A | 20460372 |
| RIPLEM | 6 | N/A | MMTV-PyMT mouse | N/A | 20460372 |
| QFDEPR | 6 | N/A | MMTV-PyMT mouse | N/A | 20460372 |
| TSAVRT | 6 | N/A | MMTV-PyMT mouse | N/A | 20460372 |
| GLWQGP | 6 | N/A | MMTV-PyMT mouse | N/A | 20460372 |
| QCTGRF | 6 | N/A | MMTV-PyMT mouse | N/A | 20460372 |
| LPGMMG | 6 | N/A | MMTV-PyMT mouse | N/A | 20460372 |
| DVGTTE | 6 | N/A | MMTV-PyMT mouse | N/A | 20460372 |
| TDLGAM | 6 | N/A | MMTV-PyMT mouse | N/A | 20460372 |
| DSNAES | 6 | N/A | MMTV-PyMT mouse | N/A | 20460372 |
| ITDMAA | 6 | N/A | MMTV-PyMT mouse | N/A | 20460372 |
| WRPCES | 6 | N/A | MMTV-PyMT mouse | N/A | 20460372 |
| WRNTIA | 6 | N/A | MMTV-PyMT mouse | N/A | 20460372 |
| IDKQLE | 6 | N/A | MMTV-PyMT mouse | N/A | 20460372 |

**Table S3.** Breast cancer specific peptides reported in the literature, with amino acid sequence, sequence size, breast cancer stage, cell line targeted, cancer histological subtype and PubMed unique identifier number (PMID) (continuation)

| **Peptide Sequence** | **Size** | **Stage** | **Cell Line** | **Histological subtype** | **PMID** |
| --- | --- | --- | --- | --- | --- |
| FMEIET | 6 | N/A | MMTV-PyMT mouse | N/A | 20460372 |
| HEVVAG | 6 | N/A | MMTV-PyMT mouse | N/A | 20460372 |
| GGHTRQ | 6 | N/A | MMTV-PyMT mouse | N/A | 20460372 |
| INGKVT | 6 | N/A | MMTV-PyMT mouse | N/A | 20460372 |
| VPWXEPAYQRFL | 12 | IV | MCF-7 | Adenocarcinoma | 20799711 |
| GRDS | 4 | IV | MCF-7 | Adenocarcinoma | 20799711 |
| RGEPAYQRFL | 10 | IV | MCF-7 | Adenocarcinoma | 20799711 |
| RGDPAYQRFL | 10 | IV | MCF-7 | Adenocarcinoma | 20799711 |
| WXEPAYQGRFL | 11 | IV | MCF-7 | Adenocarcinoma | 20799711 |
| WXEPAYNGRFL | 11 | IV | MCF-7 | Adenocarcinoma | 20799711 |
| RGEPAYQGRFL | 11 | IV | MCF-7 | Adenocarcinoma | 20799711 |
| RGDPAYQGRFL | 11 | IV | MCF-7 | Adenocarcinoma | 20799711 |
| RGEPAYNGRFL | 11 | IV | MCF-7 | Adenocarcinoma | 20799711 |
| RGDPAYNGRFL | 11 | IV | MCF-7 | Adenocarcinoma | 20799711 |
| AXEPAYQRFL | 10 | IV | MCF-7 | Adenocarcinoma | 20799711 |
| WAEPAYQRFL | 10 | IV | MCF-7 | Adenocarcinoma | 20799711 |
| WXAPAYQRFL | 10 | IV | MCF-7 | Adenocarcinoma | 20799711 |
| WXEAAYQRFL | 10 | IV | MCF-7 | Adenocarcinoma | 20799711 |
| WXEPAAQRFL | 10 | IV | MCF-7 | Adenocarcinoma | 20799711 |
| WXEPAYARFL | 10 | IV | MCF-7 | Adenocarcinoma | 20799711 |
| WXEPAYQAFL | 10 | IV | MCF-7 | Adenocarcinoma | 20799711 |
| WXEPAYQAAL | 10 | IV | MCF-7 | Adenocarcinoma | 20799711 |
| WXEPAYQAFA | 10 | IV | MCF-7 | Adenocarcinoma | 20799711 |
| EXEPAYQRFL | 10 | IV | MCF-7 | Adenocarcinoma | 20799711 |
| LXEPAYQRFL | 10 | IV | MCF-7 | Adenocarcinoma | 20799711 |
| KXEPAYQRFL | 10 | IV | MCF-7 | Adenocarcinoma | 20799711 |
| QXEPAYQRFL | 10 | IV | MCF-7 | Adenocarcinoma | 20799711 |
| YXEPAYQRFL | 10 | IV | MCF-7 | Adenocarcinoma | 20799711 |

**Table S3.** Breast cancer specific peptides reported in the literature, with amino acid sequence, sequence size, breast cancer stage, cell line targeted, cancer histological subtype and PubMed unique identifier number (PMID) (continuation)

| **Peptide Sequence** | **Size** | **Stage** | **Cell Line** | **Histological subtype** | **PMID** |
| --- | --- | --- | --- | --- | --- |
| FXEPAYQRFL | 10 | IV | MCF-7 | Adenocarcinoma | 20799711 |
| WEEPAYQRFL | 10 | IV | MCF-7 | Adenocarcinoma | 20799711 |
| WLEPAYQRFL | 10 | IV | MCF-7 | Adenocarcinoma | 20799711 |
| WIEPAYQRFL | 10 | IV | MCF-7 | Adenocarcinoma | 20799711 |
| WKEPAYQRFL | 10 | IV | MCF-7 | Adenocarcinoma | 20799711 |
| WQEPAYQRFL | 10 | IV | MCF-7 | Adenocarcinoma | 20799711 |
| WTEPAYQRFL | 10 | IV | MCF-7 | Adenocarcinoma | 20799711 |
| WXEPAYQREL | 10 | IV | MCF-7 | Adenocarcinoma | 20799711 |
| WXEPAYQRLL | 10 | IV | MCF-7 | Adenocarcinoma | 20799711 |
| WXEPAYQRKL | 10 | IV | MCF-7 | Adenocarcinoma | 20799711 |
| WXEPAYQRQ | 9 | IV | MCF-7 | Adenocarcinoma | 20799711 |
| WXEPAYQRYL | 10 | IV | MCF-7 | Adenocarcinoma | 20799711 |
| WXEPAYQRRE | 10 | IV | MCF-7 | Adenocarcinoma | 20799711 |
| WXEPAYQRFL | 10 | IV | MCF-7 | Adenocarcinoma | 20799711 |
| WXEPAYQRFK | 10 | IV | MCF-7 | Adenocarcinoma | 20799711 |
| WXEPAYQRFQ | 10 | IV | MCF-7 | Adenocarcinoma | 20799711 |
| WXEPAYQRFT | 10 | IV | MCF-7 | Adenocarcinoma | 20799711 |
| WXEPAYQRFF | 10 | IV | MCF-7 | Adenocarcinoma | 20799711 |
| XEPAYQRFL | 9 | IV | MCF-7 | Adenocarcinoma | 20799711 |
| EEPAYQRFL | 9 | IV | MCF-7 | Adenocarcinoma | 20799711 |
| LEPAYQRFL | 9 | IV | MCF-7 | Adenocarcinoma | 20799711 |
| KEPAYQRFL | 9 | IV | MCF-7 | Adenocarcinoma | 20799711 |
| QEPAYQRFL | 9 | IV | MCF-7 | Adenocarcinoma | 20799711 |
| TEPAYQRFL | 9 | IV | MCF-7 | Adenocarcinoma | 20799711 |
| XEPAYQREL | 9 | IV | MCF-7 | Adenocarcinoma | 20799711 |
| XEPAYQRLL | 9 | IV | MCF-7 | Adenocarcinoma | 20799711 |
| XEPAYQRKL | 9 | IV | MCF-7 | Adenocarcinoma | 20799711 |
| XEPAYQRQL | 9 | IV | MCF-7 | Adenocarcinoma | 20799711 |

**Table S3.** Breast cancer specific peptides reported in the literature, with amino acid sequence, sequence size, breast cancer stage, cell line targeted, cancer histological subtype and PubMed unique identifier number (PMID) (continuation)

| **Peptide Sequence** | **Size** | **Stage** | **Cell Line** | **Histological subtype** | **PMID** |
| --- | --- | --- | --- | --- | --- |
| XEPAYQRYL | 9 | IV | MCF-7 | Adenocarcinoma | 20799711 |
| XEPAYQRFL | 9 | IV | MCF-7 | Adenocarcinoma | 20799711 |
| XEPAYQRFE | 9 | IV | MCF-7 | Adenocarcinoma | 20799711 |
| XEPAYQRFL | 9 | IV | MCF-7 | Adenocarcinoma | 20799711 |
| XEPAYQRFK | 9 | IV | MCF-7 | Adenocarcinoma | 20799711 |
| XEPAYQRFQ | 9 | IV | MCF-7 | Adenocarcinoma | 20799711 |
| XEPAYQRFT | 9 | IV | MCF-7 | Adenocarcinoma | 20799711 |
| XEPAYQRFF | 9 | IV | MCF-7 | Adenocarcinoma | 20799711 |
| GGCLQILPTLSECFGR | 16 | IV | ZR-75-1 | Invasive ductal carcinoma | 16448666 |
| GLKVCGRYPGICDGIR | 16 | IV | ZR-75-1 | Invasive ductal carcinoma | 16448666 |
| GKYTWYGYSLRANWMR | 16 | IV | ZR-75-1 | Invasive ductal carcinoma | 16448666 |
| VPCQKRPGWVCLW | 13 | IV | ZR-75-1 | Invasive ductal carcinoma | 16448666 |
| KWCVIWSKEGCLF | 13 | IV | ZR-75-1 | Invasive ductal carcinoma | 16448666 |
| SSWCMRGQYNKICMW | 15 | IV | ZR-75-1 | Invasive ductal carcinoma | 16448666 |
| VECYLIRDNLCIY | 13 | IV | ZR-75-1 | Invasive ductal carcinoma | 16448666 |
| WWCLGERVVRCAH | 13 | IV | ZR-75-1 | Invasive ductal carcinoma | 16448666 |
| FYCVIERLGVCLY | 13 | IV | ZR-75-1 | Invasive ductal carcinoma | 16448666 |
| RVCFLWQDGRCVF | 13 | IV | ZR-75-1 | Invasive ductal carcinoma | 16448666 |
| HKHGHGHGKHKNKGK | 15 | IV | MDA-MB-231 | Adenocarcinoma | 14506238 |
| KHGHGHGK | 8 | IV | MDA-MB-231 | Adenocarcinoma | 14506238 |
| KGHHGKHG | 8 | IV | MDA-MB-231 | Adenocarcinoma | 14506238 |
| HKNKGKKN | 8 | IV | MDA-MB-231 | Adenocarcinoma | 14506238 |
| LTVSPLWD | 8 | IV | SK-BR-3 | Adenocarcinoma | 12490548 |
| NLYYASW | 7 | IV | SK-BR-3 | Adenocarcinoma | 12490548 |
| MARSGL | 6 | IV | SK-BR-3, MDA-MB-468 | Adenocarcinoma | 11340582 |
| MARAKE | 6 | IV | SK-BR-3, MDA-MB-468 | Adenocarcinoma | 11340582 |
| MSRTMS | 6 | IV | SK-BR-3, MDA-MB-468 | Adenocarcinoma | 11340582 |
| AEGEFMYWGDSHWLQYWYEGDPAK | 24 | IV | SK-BR-3 | Adenocarcinoma | 11700053 |

**Table S3.** Breast cancer specific peptides reported in the literature, with amino acid sequence, sequence size, breast cancer stage, cell line targeted, cancer histological subtype and PubMed unique identifier number (PMID) (continuation)

| **Peptide Sequence** | **Size** | **Stage** | **Cell Line** | **Histological subtype** | **PMID** |
| --- | --- | --- | --- | --- | --- |
| AEGEFWGDSHWLQYWYEGDPAK | 22 | IV | SK-BR-3 | Adenocarcinoma | 11700053 |
| AEGEFIHNRYNRFFYWYGDPAK | 22 | IV | SK-BR-3 | Adenocarcinoma | 11700053 |
| AEGEFPRWGDSHWLQYWYEGDPAK | 24 | IV | SK-BR-3 | Adenocarcinoma | 11700053 |
| AEGEFLMWGGSHWLEYWYEGDPAK | 24 | IV | SK-BR-3 | Adenocarcinoma | 11700053 |
| AEGEFGHWCDQHWLQYWYEGDPAK | 24 | IV | SK-BR-3 | Adenocarcinoma | 11700053 |
| AEGEFGWWGDSHWLQYEGDPAK | 22 | IV | SK-BR-3 | Adenocarcinoma | 11700053 |
| CRGDKGPDC | 9 | IV | BT-474, MDA-MB-231, 4T1 (mouse tumor model) | Invasive Ductal Carcinoma, Adenocarcinoma | 19962669 |
| KGVSLSYRKKGVSLSYR | 17 | IV | MDA-MB-231 | Adenocarcinoma | 19482312 |
| QEFSPYMGLEFKKH | 14 | IV | MCF-7 | Adenocarcinoma | 15722342 |
| QEFSPNLWGLEFQKN | 15 | IV | MCF-7 | Adenocarcinoma | 15722342 |
| QEYSPNLWGHEFRSH | 15 | IV | MCF-7 | Adenocarcinoma | 15722342 |
| QHWSYKCIRP | 10 | IV | MCF-7 | Adenocarcinoma | 16123131 |
| CRGDK | 5 | IV | MDA-MB-321 | Adenocarcinoma | 22056754 |
| KDEPQRRSARLSAKPAPPKPEPKPKKAPAKK | 31 | IV | MDA-MB-231 | Adenocarcinoma | 22052706 |
| CGQKRTRGC | 9 | IV | MDA-MB-231 | Adenocarcinoma | 22052706 |
| HPLSKHPYWSQP | 12 | N/A | 4T1 (mouse tumor model) | N/A | 22119929 |
| APRPG | 5 | IV | MCF-7 | Adenocarcinoma | 21904456 |
| CGKRK | 5 | IV | MDA-MB-231 | Adenocarcinoma | 21739966 |
| CSNIDARAC | 9 | IV, III | MDA-MB-231, HT-29 | Adenocarcinoma | 21222482 |
| CDPSRGKNC | 9 | IV, III | MDA-MB-231, HT-29 | Adenocarcinoma | 21222482 |
| CPSDLKDAC | 9 | IV, III | MDA-MB-231, HT-29 | Adenocarcinoma | 21222482 |
| CRTTRGTKC | 9 | IV, III | MDA-MB-231, HT-29 | Adenocarcinoma | 21222482 |
| CRMTRNKPC | 9 | IV, III | MDA-MB-231, HT-29 | Adenocarcinoma | 21222482 |
| CRVSRQNKC | 9 | IV, III | MDA-MB-231, HT-29 | Adenocarcinoma | 21222482 |
| CAKIDPELC | 9 | IV, III | MDA-MB-231, HT-29 | Adenocarcinoma | 21222482 |
| CGGERGKSC | 9 | IV, III | MDA-MB-231, HT-29 | Adenocarcinoma | 21222482 |
| YIEGLQALLRDQ | 12 | IV, III | MCF-7, HT-29 | Adenocarcinoma | NA |
| IFLLWQR | 7 | IV, I | MDA-MB-231, HCT-116 | Adenocarcinoma, Colorectal carcinoma | 22114188 |
