# Supplementary material for: Screening and characterization of novel specific peptides targeting MDA-MB-231 claudin-low breast carcinoma by computer-aided phage display methodologies
Source: BMC Cancer. 2016 Nov 14;16:881. doi: 10.1186/s12885-016-2937-2 (PMC5109716; doi:10.1186/s12885-016-2937-2)
Supplement: Additional file 3: Table S4. — Data collected for potential biomarkers of breast cancer cells, retrieved from Kyoto Encyclopedia of Genes and Genomes (KEGG), Uniprot, GenBank, and Protein Data Bank (PDB), with those present in MDA-MB-231 represented in bold. (DOCX 27 kb) [file 12885_2016_2937_MOESM3_ESM.docx]

Additional file 3: **Table S4.** Data collected for potential biomarkers of breast cancer cells, retrieved from Kyoto Encyclopedia of Genes and Genomes (KEGG), Uniprot, GenBank, and Protein Data Bank (PDB), with those present in MDA-MB-231 represented in bold

| **Biomarkers** | **KEGG** | **Uniprot** | **GenBank** | **PDB** |
| --- | --- | --- | --- | --- |
| **Alpha-1-antichymotrypsin** | hsa:12 | P01011 | NC_000014.9 | 3DLW |
| **Annexin V** | hsa:308 | P08758 | NC_000004.12 | 1avr |
| **BAG-1** | hsa:573 | Q99933 | NC_000009.12 | N/A |
| **Apoptosis regulator Bcl-2** | hsa:596 | P10415 | NC_000018.10 | 1g5m |
| **C-C chemokine receptor type 7 (CCR7)** | hsa:1236 | P32248 | NC_000017.11 | N/A |
| **C-X-C chemokine receptor type 4 (CXCR4)** | hsa:7852 | P61073 | NC_000002.12 | N/A |
| **Cystatin-SAIII** | hsa:1472 | P01036 | NC_000020.11 | N/A |
| **Elafin** | hsa:5266 | P19957 | NC_000020.11 | N/A |
| **Enolase 1** | hsa:2023 | P06733 | NC_000001.11 | N/A |
| **Galectin-1** | hsa:3956 | P09382 | NC_000022.11 | N/A |
| **Galectin-3-binding protein** | hsa:3959 | Q08380 | NC_000017.11 | N/A |
| **Glucose regulated protein 78 or heat shock protein 5 (GRP78)** | hsa:3309 | P11021 | NC_000009.12 | N/A |
| **Heat-shock protein HSP90A** | hsa:3320 | P07900 | NC_000014.9 | N/A |
| **Kallikrein-5 (KLK5)** | hsa:25818 | Q9Y337 | NC_000019.10 | N/A |
| **Lysyl oxidase homolog 2 precursor (LOXL2)** | hsa:4017 | Q9Y4K0 | NC_000008.11 | N/A |
| **Mesothelin isoform 1** | hsa:10232 | Q13421 | NC_000016.10 | N/A |
| **Metalloproteinase inhibitor 1 (TIMP-1)** | hsa:7076 | P01033 | NC_000023.11 | N/A |
| **Matrix metalloproteinase-26 (MMP-26)** | hsa:56547 | Q9NRE1 | NC_000011.10 | N/A |
| **Matrix metalloproteinase-9 (MMP-9)** | hsa:4318 | P14780 | NC_000020.11 | N/A |
| **Cellular tumor antigen p53** | hsa:7157 | P04637 | NC_000017.11 | 1A1U |
| **Plasminogen activator inhibitor 1 precursor (PAI1)** | hsa:5054 | P05121 | NC_000007.14 | N/A |
| **Peptidyl-prolyl cis-trans isomerase A (Pin1)** | hsa:5300 | Q13526 | NC_000019.10 | N/A |

**Table S4.** Data collected for potential biomarkers of breast cancer cells, retrieved from Kyoto Encyclopedia of Genes and Genomes (KEGG), Uniprot, GenBank, and Protein Data Bank (PDB), with those present in MDA-MB-231 represented in bold (continuation)

| **Biomarkers** | **KEGG** | **Uniprot** | **GenBank** | **PDB** |
| --- | --- | --- | --- | --- |
| **Synuclein-γ (SNCG)** | hsa:6623 | Q6FHG5 | NC_000010.11 | N/A |
| **Thrombospondin-1 (TSP-1)** | hsa:7057 | P07996 | NC_000015.10 | N/A |
| **Ubiquitin-conjugating enzyme E2 C (UBE2C)** | hsa:11065 | O00762 | NC_000020.11 | 1i7k |
| **α-Tubulin** | hsa:7846 | Q71U36 | NC_000012.12 | N/A |
