# Supplementary material for: Screening and characterization of novel specific peptides targeting MDA-MB-231 claudin-low breast carcinoma by computer-aided phage display methodologies
Source: BMC Cancer. 2016 Nov 14;16:881. doi: 10.1186/s12885-016-2937-2 (PMC5109716; doi:10.1186/s12885-016-2937-2)
Supplement: Additional file 4: Figure S1. — Flow cytometry results, in terms of percentage of binding, of the phage pool from the last round of 12-mer conventional panning against normal breast cell line MCF-10-2A, breast cancer cell lines MDA-MB-231, SK-BR-3, Hs 578 T and MDA-MB-435 cell line [19]. Statistically significant (P) differences are represented by ***. (DOCX 43 kb) [file 12885_2016_2937_MOESM4_ESM.docx]

Additional file 4

**Flow cytometry analysis**


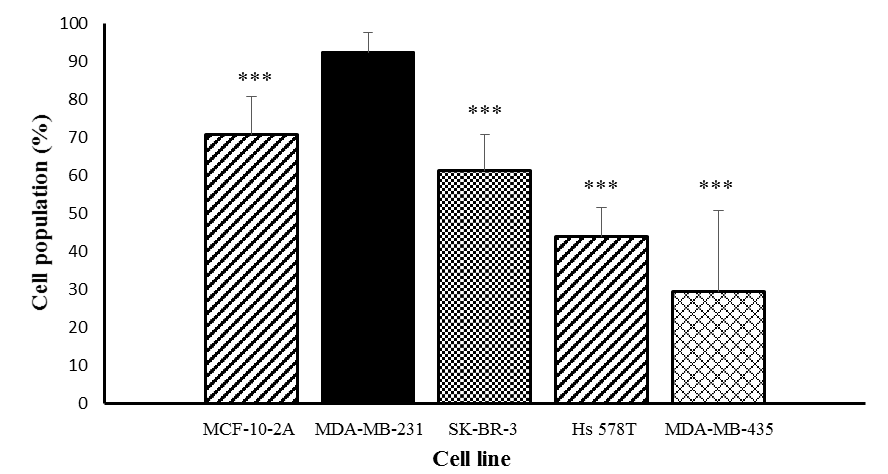


**Figure S1.** Flow cytometry results, in terms of percentage of binding, of the phage pool from the last round of 12-mer conventional panning against normal breast cell line MCF-10-2A, breast cancer cell lines MDA-MB-231, SK-BR-3, Hs 578T and MDA-MB-435 cell line [19]. Statistically significant (*P*) differences are represented by ***
