# Supplementary material for: Screening and characterization of novel specific peptides targeting MDA-MB-231 claudin-low breast carcinoma by computer-aided phage display methodologies
Source: BMC Cancer. 2016 Nov 14;16:881. doi: 10.1186/s12885-016-2937-2 (PMC5109716; doi:10.1186/s12885-016-2937-2)
Supplement: Additional file 5: Figure S2. — Immunofluorescence staining of MDA-MB-231 tissue sections. Sections were incubated with (A) wild-type M13KE phage particles and (B) M13KE phage particles of the phage pool from the last round of conventional phage display. Images were acquired with blue filter (1), green filter (2) and filter overlapping (3). Peptide affinity was detected using a primary anti-M13 antibody and a secondary goat anti-rabbit FITC conjugate antibody. Images were acquired using an inverted LEICA DMI 3000B (Leica Mycrosystems) with incorporated camera (Model DFC 450C). Scale bar of 10 μm. (DOCX 1254 kb) [file 12885_2016_2937_MOESM5_ESM.docx]

Additional file 5

**Tissue section analysis**


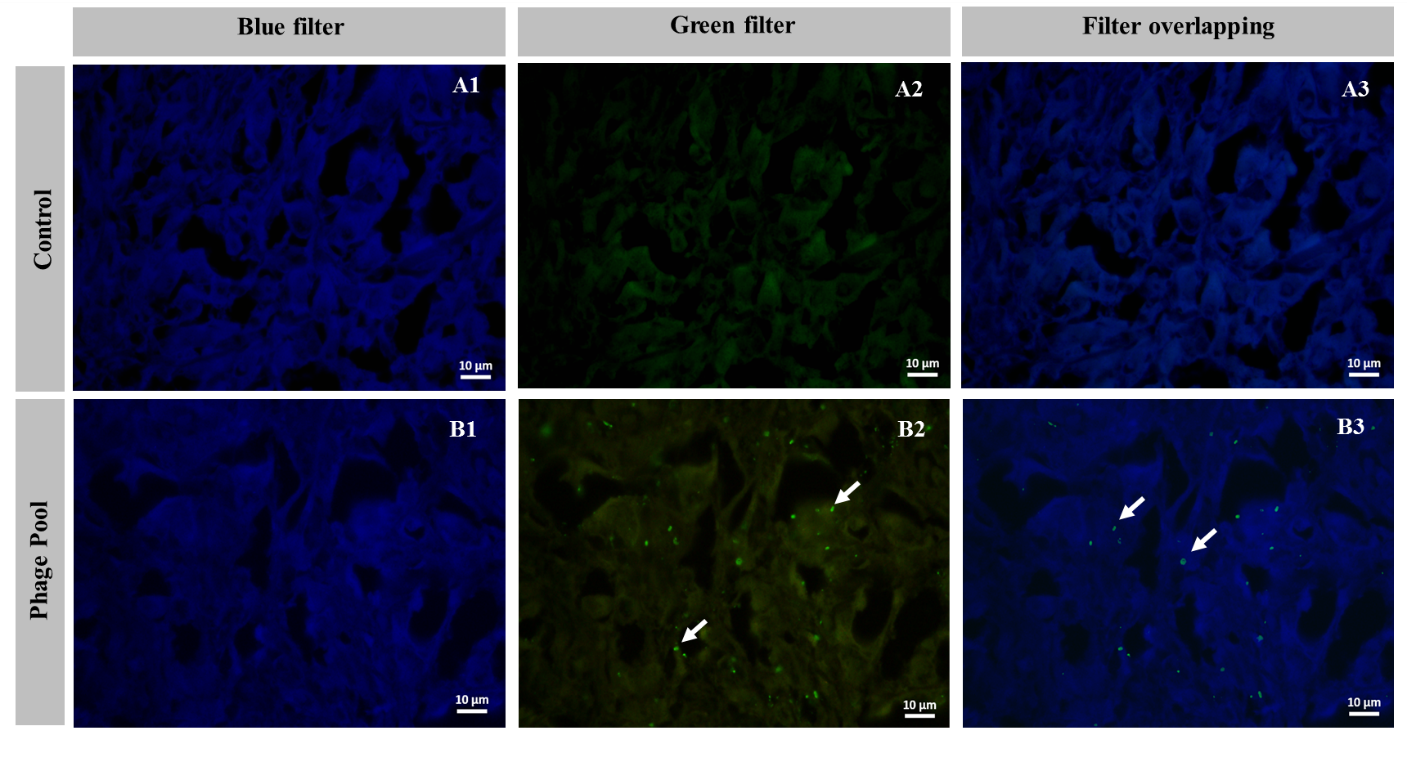


**Figure S2.** Immunofluorescence staining of MDA-MB-231 tissue sections. Sections were incubated with (A) wild-type M13KE phage particles and (B) M13KE phage particles of the phage pool from the last round of conventional phage display. Images were acquired with blue filter (1), green filter (2) and filter overlapping (3). Peptide affinity was detected using a primary anti-M13 antibody and a secondary goat anti-rabbit FITC conjugate antibody. Images were acquired using an inverted LEICA DMI 3000B (Leica Mycrosystems) with incorporated camera (Model DFC 450C). Scale bar of 10 µm
