# Supplementary material for: Screening and characterization of novel specific peptides targeting MDA-MB-231 claudin-low breast carcinoma by computer-aided phage display methodologies
Source: BMC Cancer. 2016 Nov 14;16:881. doi: 10.1186/s12885-016-2937-2 (PMC5109716; doi:10.1186/s12885-016-2937-2)
Supplement: Additional file 6: Figure S3. — Heatmap representation of the similarities between all peptides identified in this work with those previously reported. New12Br: 12-mer peptides obtained in this work using the BRASIL methodology; New12Conv: 12-mer peptides obtained in this work using the conventional methodology; Previous: 12-mer peptides reported in previous studies. Legend bar on the right represents the peptides of x-mer in different colours. (DOCX 398 kb) [file 12885_2016_2937_MOESM6_ESM.docx]

Additional file 6

**Bioinformatics analysis**

**
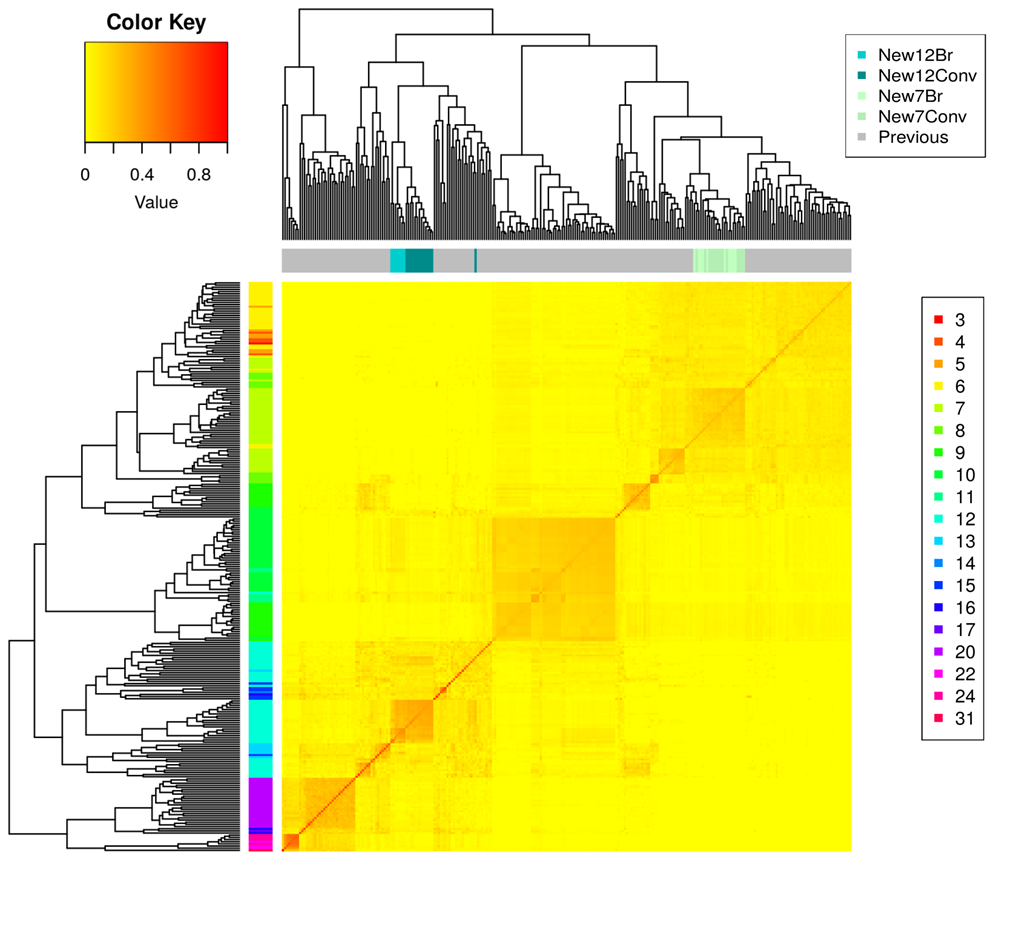
**

**Figure S3.** Heatmap representation of the similarities between all peptides identified in this work with those previously reported. New12Br: 12-mer peptides obtained in this work using the BRASIL methodology; New12Conv: 12-mer peptides obtained in this work using the conventional methodology; Previous: 12-mer peptides reported in previous studies. Legend bar on the right represents the peptides of x-mer in different colours
