# Supplementary material for: Screening and characterization of novel specific peptides targeting MDA-MB-231 claudin-low breast carcinoma by computer-aided phage display methodologies
Source: BMC Cancer. 2016 Nov 14;16:881. doi: 10.1186/s12885-016-2937-2 (PMC5109716; doi:10.1186/s12885-016-2937-2)
Supplement: Additional file 7: Table S2. — Data from docking analysis with the selected 7-mer and 12-mer phage display peptides against breast cancer biomarkers retrieved from the literature: lowest energy weighted score (E), cluster members (CM) and type of interaction (I). Biomarkers present in MDA-MB-231 cells and best model scores are given in bold. (DOCX 41 kb) [file 12885_2016_2937_MOESM7_ESM.docx]

Additional file 7

**Table S2.** Data from docking analysis with the selected 7-mer and 12-mer phage display peptides against breast cancer biomarkers retrieved from the literature: lowest energy weighted score (*E*), cluster members (CM) and type of interaction (I). Biomarkers present in MDA-MB-231 cells and best model scores are given in bold

| **BREAST CANCER BIOMARKER** | **PEPTIDE** | | | | | | | | | | | | | | |
| --- | --- | --- | --- | --- | --- | --- | --- | --- | --- | --- | --- | --- | --- | --- | --- |
|  | **1.3 (7/52)** | | | **5.3 (14/45)** | | | **5.3 (19/45)** | | | **6.2 (8/17)** | | | **6.2 (9/17)** | | |
|  | ***E^a^*** | **CM^b^** | **I^c^** | ***E*** | **CM** | **I** | ***E*** | **CM** | **I** | ***E*** | **CM** | **I** | ***E*** | **CM** | **I** |
| **Alpha-1-antichymotrypsin** | -1012 | 338 | Hydrophobic | -861 | 412 | Balanced | -1050 | 277 | Electrostatic | -999 | 358 | Hydrophobic | -935 | 326 | Hydrophobic |
| **Annexin V** | -1029 | 543 | Hydrophobic | -879 | 300 | Electrostatic | -1487 | 438 | Hydrophobic | -984 | 524 | Hydrophobic | -1009 | 538 | Hydrophobic |
| **BAG-1** | -1094 | 354 | Hydrophobic | -1230 | 217 | Hydrophobic | -868 | 231 | Electrostatic | -909 | 430 | Hydrophobic | -860 | 585 | Hydrophobic |
| **Apoptosis regulator Bcl-2** | -1087 | 350 | Hydrophobic | -1223 | 180 | Hydrophobic | -1434 | 183 | Hydrophobic | -906 | 212 | Hydrophobic | -921 | 386 | Hydrophobic |
| **C-C chemokine receptor type 7 (CCR7)** | -1347 | 237 | Hydrophobic | -1788 | 242 | Hydrophobic | -2037 | 261 | Hydrophobic | -1415 | 238 | Hydrophobic | -955 | 330 | Balanced |
| **C-X-C chemokine receptor type 4 (CXCR4)** | -1602 | 542 | Hydrophobic | -2023 | 354 | Hydrophobic | -2436 | 528 | Hydrophobic | -919 | 156 | Balanced | -1379 | 221 | Hydrophobic |
| **Cystatin-SAIII** | -1118 | 505 | Hydrophobic | -829 | 259 | Balanced | -1386 | 216 | Hydrophobic | -950 | 317 | Hydrophobic | -977 | 496 | Hydrophobic |
| **Elafin** | -721 | 339 | Balanced | -1122 | 238 | Hydrophobic | -1302 | 212 | Hydrophobic | -873 | 372 | Hydrophobic | -902 | 326 | Hydrophobic |
| **Enolase 1** | -931 | 331 | Hydrophobic | -1011 | 123 | Hydrophobic | -1364 | 167 | Hydrophobic | -843 | 281 | Hydrophobic | -924 | 349 | Hydrophobic |
| **Galectin-1** | -913 | 543 | Hydrophobic | -933 | 199 | Hydrophobic | -1207 | 282 | Hydrophobic | -812 | 289 | Hydrophobic | -823 | 372 | Hydrophobic |
| **Galectin-3-binding protein** | -1202 | 363 | Hydrophobic | -1591 | 326 | Hydrophobic | -2022 | 340 | Hydrophobic | -1068 | 494 | Hydrophobic | -819 | 586 | Electrostatic |
| **Glucose regulated protein 78 or heat shock protein 5 (GRP78)** | -768 | 302 | Balanced | -914 | 255 | Balanced | -1471 | 403 | Hydrophobic | -1025 | 480 | Hydrophobic | -868 | 442 | Hydrophobic |
| **Heat-shock protein HSP90A** | -1433 | 453 | Hydrophobic | -1235 | 318 | Electrostatic | -2102 | 352 | Hydrophobic | -1076 | 331 | Electrostatic | -1269 | 275 | Hydrophobic |
| **Kallikrein-5 (KLK5)** | -824 | 326 | Balanced | -1624 | 282 | Hydrophobic | -1779 | 492 | Hydrophobic | -1160 | 388 | Hydrophobic | -1096 | 326 | Hydrophobic |
| **Lysyl oxidase homolog 2 precursor (LOXL2)** | -1124 | 225 | Hydrophobic | -1281 | 187 | Hydrophobic | -1473 | 253 | Hydrophobic | -968 | 563 | Hydrophobic | -871 | 184 | Hydrophobic |
| **Mesothelin isoform 1** | -1368 | 426 | Hydrophobic | -1559 | 322 | Hydrophobic | -1724 | 235 | Hydrophobic | -809 | 243 | Electrostatic | -1066 | 550 | Hydrophobic |
| **Metalloproteinase inhibitor 1 (TIMP-1)** | **-1127** | **595** | Hydrophobic | -1452 | 193 | Hydrophobic | -1603 | 222 | Hydrophobic | -811 | 294 | Balanced | -1144 | 404 | Hydrophobic |
| **Matrix metalloproteinase-26 (MMP-26)** | -1261 | 385 | Hydrophobic | -1199 | 230 | Electrostatic | -1782 | 240 | Hydrophobic | -1208 | 497 | Hydrophobic | -1227 | 311 | Hydrophobic |
| **Matrix metalloproteinase-9 (MMP-9)** | -1086 | 317 | Balanced | -1696 | 202 | Hydrophobic | -1852 | 198 | Hydrophobic | -1183 | 235 | Hydrophobic | -1165 | 424 | Hydrophobic |

**Table S2.** Data from docking analysis with the selected 7-mer and 12-mer phage display peptides against breast cancer biomarkers retrieved from the literature: lowest energy weighted score (*E*), cluster members (CM) and type of interaction (I). Biomarkers present in MDA-MB-231 cells and best model scores are given in bold (continuation)

| **BREAST CANCER BIOMARKER** | **PEPTIDE** | | | | | | | | | | | | | | |
| --- | --- | --- | --- | --- | --- | --- | --- | --- | --- | --- | --- | --- | --- | --- | --- |
|  | **1.3 (7/52)** | | | **5.3 (14/45)** | | | **5.3 (19/45)** | | | **6.2 (8/17)** | | | **6.2 (9/17)** | | |
|  | ***E^a^*** | **CM^b^** | **I^c^** | ***E*** | **CM** | **I** | ***E*** | **CM** | **I** | ***E*** | **CM** | **I** | ***E*** | **CM** | **I** |
| **Cellular tumor antigen p53** | -760 | 233 | Balanced | -1148 | 336 | Balanced | -1212 | 365 | Electrostatic | -805 | 328 | Electrostatic | -969 | 256 | Hydrophobic |
| **Plasminogen activator inhibitor 1 (PAI1)** | -1402 | 481 | Hydrophobic | -870 | 224 | Balanced | -1722 | 370 | Hydrophobic | -1046 | 318 | Hydrophobic | **-1047** | **741** | **Hydrophobic** |
| **Peptidyl-prolyl cis-trans isomerase A (Pin1)** | -969 | 432 | Hydrophobic | -1109 | 203 | Hydrophobic | -1025 | 255 | Electrostatic | -965 | 415 | Hydrophobic | -912 | 582 | Hydrophobic |
| **Synuclein-γ (SNCG)** | -533 | 318 | Electrostatic | -869 | 211 | Hydrophobic | -1025 | 453 | Hydrophobic | -535 | 396 | Balanced | -636 | 219 | Hydrophobic |
| **Thrombospondin-1 (TSP-1)** | -1148 | 440 | Hydrophobic | -1208 | 322 | Electrostatic | -1305 | 203 | Electrostatic | -995 | 159 | Hydrophobic | -1016 | 254 | Hydrophobic |
| **Ubiquitin-conjugating enzyme E2 C (UBE2C)** | -1072 | 312 | Hydrophobic | -1182 | 118 | Hydrophobic | -965 | 252 | Balanced | -676 | 322 | Balanced | -852 | 361 | Hydrophobic |
| **α-Tubulin** | -1211 | 391 | Hydrophobic | -1375 | 278 | Hydrophobic | -1900 | 318 | Hydrophobic | -1064 | 482 | Hydrophobic | -894 | 278 | Hydrophobic |
| **β-Actin** | -1149 | 598 | Hydrophobic | -1599 | 316 | Hydrophobic | **-1697** | **600** | **Hydrophobic** | **-1129** | **661** | **Hydrophobic** | -1103 | 498 | Hydrophobic |
| **β-Dystroglycan precursor** | -1107 | 281 | Hydrophobic | -938 | 238 | Electrostatic | -1594 | 194 | Hydrophobic | -934 | 173 | Hydrophobic | -933 | 216 | Hydrophobic |
| Alpha-1-antitrypsin | -685 | 277 | Electrostatic | -1141 | 274 | Hydrophobic | -1024 | 247 | Balanced | -767 | 262 | Hydrophobic | -755 | 401 | Hydrophobic |
| Carcinoembryonic antigen (CEA) | -835 | 502 | Balanced | -1442 | 193 | Hydrophobic | -1074 | 165 | Electrostatic | -814 | 197 | Electrostatic | -1027 | 215 | Hydrophobic |
| Receptor tyrosine-protein kinase erbB-2 | -1294 | 234 | Hydrophobic | -1140 | 207 | Electrostatic | -1862 | 325 | Hydrophobic | -1050 | 154 | Hydrophobic | -1154 | 446 | Hydrophobic |
| Clusterin precursor | -628 | 548 | Balanced | -870 | 245 | Hydrophobic | -1147 | 271 | Hydrophobic | -642 | 497 | Electrostatic | -743 | 540 | Hydrophobic |
| cytokeratin-18 | -948 | 521 | Hydrophobic | -1116 | 159 | Hydrophobic | -1242 | 373 | Hydrophobic | -845 | 353 | Hydrophobic | -964 | 652 | Hydrophobic |
| E-cadherin | -978 | 292 | Hydrophobic | **-1106** | **443** | **Electrostatic** | -1447 | 287 | Hydrophobic | -960 | 260 | Hydrophobic | -872 | 22 | Hydrophobic |
| Kallikrein-10 (KLK10) | -1163 | 423 | Hydrophobic | -1130 | 277 | Balanced | -1941 | 428 | Hydrophobic | -1354 | 498 | Hydrophobic | -1226 | 530 | Hydrophobic |
| Kallikrein-6 (KLK6) | -741 | 268 | Electrostatic | -1263 | 225 | Hydrophobic | -929 | 324 | Electrostatic | -881 | 204 | Hydrophobic | -860 | 258 | Hydrophobic |
| Mesothelin isoform 1 preproprotein | -985 | 631 | Hydrophobic | -1029 | 274 | Hydrophobic | -1255 | 265 | Hydrophobic | -744 | 307 | Hydrophobic | -734 | 501 | Hydrophobic |
| Nucleoside diphosphate kinase A (nm23-H1) | -807 | 217 | Electrostatic | -1402 | 138 | Hydrophobic | -1625 | 214 | Hydrophobic | -822 | 150 | Electrostatic | -966 | 143 | Hydrophobic |
| Prolactin-inducible protein (PIP) | -938 | 476 | Hydrophobic | -1084 | 247 | Hydrophobic | -1341 | 401 | Hydrophobic | -692 | 652 | Electrostatic | -633 | 342 | Electrostatic |
| Secreted protein acidic and rich in cysteine (SPARC) precursor | -1130 | 376 | Hydrophobic | -1626 | 269 | Hydrophobic | -1743 | 315 | Hydrophobic | -1200 | 458 | Hydrophobic | -1153 | 424 | Hydrophobic |

^a^ Weighted score is calculated according to formula *E* = 0.40*E*rep + −0.40*E*_att_ + 600*E*_elec_ + 1.00*E*_DARS_ (Balanced), *E* = 0.40*E*_rep_ + −0.40*E*_att_+ 1200*E*_elec_ + 1.00*E*_DARS_ (Electrostatic-favored), *E* = 0.40*E*_rep_ + −0.40*E*_att_ + 600*E*_elec_ + 2.00*E*_DARS_ (Hydrophobic-favored), or *E* = 0.40*E*_rep_ + −0.10*E*_att_ + 600*E*_elec_ + 0.00*E*_DARS_ (van der Waals and Electrostatic).

^b^ ClusPro 2.0 ranks models by cluster size. 1000 rotation/translation combinations of lowest score are chosen from 70,000 rotations performed, and are clustered together to find the ligand position with the most “neighbors” in 9 angstroms, becoming a cluster center and the neighbors the members of the cluster. A second cluster center is obtained with the remaining rotations and so on. So the most members on the cluster, the most significant the result.

^c^ Coefficient weights of *E* formula adapted for Balanced, Electrostatic-favored, Hydrophobic-favored or van der Waals and Electrostatic interactions.
